# Computer-aided design and implementation of efficient biosynthetic pathways to produce high added-value products derived from tyrosine in *Escherichia coli*

**DOI:** 10.3389/fbioe.2024.1360740

**Published:** 2024-06-24

**Authors:** Sofia Ferreira, Alexandra Balola, Anastasia Sveshnikova, Vassily Hatzimanikatis, Paulo Vilaça, Paulo Maia, Rafael Carreira, Ruth Stoney, Pablo Carbonell, Caio Silva Souza, João Correia, Diana Lousa, Cláudio M. Soares, Isabel Rocha

**Affiliations:** ^1^ Systems and Synthetic Biology Laboratory, ITQB Nova–Instituto de Tecnologia Química e Biológica António Xavier, Oeiras, Portugal; ^2^ Laboratory of Computational Systems Biotechnology, École Polytechnique Fédérale de Lausanne, EPFL, Lausanne, Switzerland; ^3^ SilicoLife–Computational Biology Solutions for the Life Sciences, Braga, Portugal; ^4^ Manchester Institute of Biotechnology, School of Chemistry, Faculty of Science and Engineering, University of Manchester, Manchester, United Kingdom; ^5^ Institute of Industrial Control Systems and Computing (AI2), Universitat Politècnica de València (UPV), Valencia, Spain; ^6^ Institute for Integrative Systems Biology I2SysBio, Universitat de València-CSIC: Consejo Superior de Investigaciones Científicas, Paterna, Spain; ^7^ Protein Modelling Laboratory, ITQB Nova—Instituto de Tecnologia Química e Biológica António Xavier, Oeiras, Portugal

**Keywords:** biosynthetic pathways, retrobiosynthesis algorithms, L-DOPA, dopamine, computational tools, bioprocesses, pathway design, *Escherichia coli*

## Abstract

Developing efficient bioprocesses requires selecting the best biosynthetic pathways, which can be challenging and time-consuming due to the vast amount of data available in databases and literature. The extension of the shikimate pathway for the biosynthesis of commercially attractive molecules often involves promiscuous enzymes or lacks well-established routes. To address these challenges, we developed a computational workflow integrating enumeration/retrosynthesis algorithms, a toolbox for pathway analysis, enzyme selection tools, and a gene discovery pipeline, supported by manual curation and literature review. Our focus has been on implementing biosynthetic pathways for tyrosine-derived compounds, specifically L-3,4-dihydroxyphenylalanine (L-DOPA) and dopamine, with significant applications in health and nutrition. We selected one pathway to produce L-DOPA and two different pathways for dopamine–one already described in the literature and a novel pathway. Our goal was either to identify the most suitable gene candidates for expression in *Escherichia coli* for the known pathways or to discover innovative pathways. Although not all implemented pathways resulted in the accumulation of target compounds, in our shake-flask experiments we achieved a maximum L-DOPA titer of 0.71 g/L and dopamine titers of 0.29 and 0.21 g/L for known and novel pathways, respectively. In the case of L-DOPA, we utilized, for the first time, a mutant version of tyrosinase from *Ralstonia solanacearum*. Production of dopamine via the known biosynthesis route was accomplished by coupling the L-DOPA pathway with the expression of DOPA decarboxylase from *Pseudomonas putida*, resulting in a unique biosynthetic pathway never reported in literature before. In the context of the novel pathway, dopamine was produced using tyramine as the intermediate compound. To achieve this, tyrosine was initially converted into tyramine by expressing TDC from *Levilactobacillus brevis*, which, in turn, was converted into dopamine through the action of the enzyme encoded by *ppoMP* from *Mucuna pruriens*. This marks the first time that an alternative biosynthetic pathway for dopamine has been validated in microbes. These findings underscore the effectiveness of our computational workflow in facilitating pathway enumeration and selection, offering the potential to uncover novel biosynthetic routes, thus paving the way for other target compounds of biotechnological interest.

## 1 Introduction

The shikimate pathway links the carbohydrate metabolism with the synthesis of aromatic amino acids, involving the formation of several aromatic intermediates, which are precursors to a great variety of secondary metabolites with applications in the pharmaceutical and food industries (Averesch and Krömer, 2018). Thus, the aromatic nature of this pathway holds a huge commercial interest with the potential for replacing fossil fuel-derived aromatics and plant-based compounds. However, the complex nature of some of these compounds combined with the lack of established pathways and known enzymes/genes impairs the development of efficient microbial cell factories when solely relying on a manual revision of databases and literature.

Of particular interest are compounds derived from tyrosine, such as L-DOPA and dopamine, which have significant industrial and commercial value as prescription drugs for the treatment of various medical conditions (Min et al., 2015; Juárez Olguín et al., 2016). Recent research has focused on producing these compounds in *Escherichia coli* to establish efficient and sustainable bioprocesses. Two types of enzymes, tyrosinase (Tyr) and *p*-hydroxyphenylacetate 3-hydroxylase (PHAH), have been investigated for the conversion of tyrosine into L-DOPA. Subsequently, L-DOPA can be converted into dopamine through the action of DOPA decarboxylase (DDC). In recent years, despite the abundance of protein candidates catalyzing these reactions, only a few of them have been explored. Maximum reported L-DOPA titers obtained with PHAH are in the 700 mg/L range (Fordjour et al., 2019), while Tyr allowed to obtain up to 300 mg/L. However, when the *ddc* gene is added, dopamine accumulation exceeded 1 g/L (Nakagawa et al., 2011).

In recent years, retrosynthetic and enumeration computational tools have emerged to harness continuously curated and updated metabolic network databases, greatly facilitating the design of novel and efficient biosynthetic pathways. The diversity in tools is associated with differences in metabolic network representation, such as the use of graphs or stoichiometric matrices, and the specific search algorithms they employ, including graph searches, flux balance analysis, or retrosynthetic searches. The selection of these tools often hinges on several factors, including the nature of the target metabolites and the preferred host organism (Wang et al., 2017; Sveshnikova et al., 2022b). After (re)constructing a specific pathway, it becomes necessary to assign an enzyme sequence to each catalytic reaction for the *in vivo* implementation. To facilitate this task, a range of bioinformatics tools is available to mine candidate protein/gene sequences. These tools employ diverse approaches, including molecular simulations, density functional theory, or partitioned quantum mechanics and molecular mechanics, or machine learning techniques (Alderson et al., 2012; Rahman et al., 2014; Mellor et al., 2016; Carbonell et al., 2018).

To address the challenges in aromatic compound production while expanding nature’s portfolio, we have established a computational workflow that combines pathway design tools based on enumeration, such as FindPath (Liu et al., 2015), and retrobiosynthesis search algorithms, in particular BNICE.ch (Hatzimanikatis et al., 2005) and RetroPath2.0 (Delépine et al., 2018); a Retrotoolbox for pathway analysis [ShikiAtlas (Mohammadi-Peyhani et al., 2021; Sveshnikova et al., 2022a)]; enzyme selection tools – BridgIT (Hadadi et al., 2019) and Selenzyme (Carbonell et al., 2018) – and an in-house gene-finding pipeline. This workflow aims to efficiently design pathways for the production of high-value tyrosine-derived compounds in *Escherichia coli*, including L-DOPA and dopamine.

From the generated pathways, we considered biochemical routes containing known enzymes or orphan reactions (which lack protein or gene sequences associated), favoring maximum carbon conservation and minimal length. BridgIT and Selenzyme were used to assign an Enzyme Commission (EC) number to each reaction and provide a first indication of appropriate gene candidates, and an in-house structure-based gene discovery pipeline ranked the most suitable gene candidates for expression in *E. coli*, focusing on prokaryotic sources to prevent issues arising from protein solubility and post-translational modification. This information was complemented with relevant data retrieved from literature and databases, giving preference to enzyme variants with enhanced activity and previously validated expression in *E. coli*. The designed pathways were then implemented *in vivo* using molecular biology methods, with various designs tested.

**FIGURE 1:**
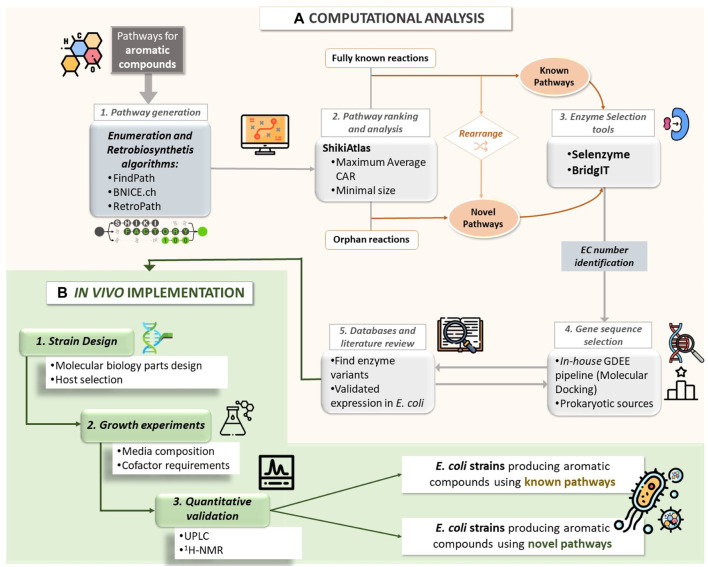
Workflow of the **(A)** computational analysis of various biosynthetic pathways to yield aromatic compounds, including the generation of pathways through retrosynthesis and enumeration algorithms favoring maximum Conserved Atom Ratio (CAR) and minimal size. For the different reactions constituting the generated pathways, the enzyme selection tools Selenzyme and BrigIT were utilized to attribute an Enzyme Commission (EC) number. This step aided in the selection of the template enzyme for performing Molecular Docking using the in-house Gene Discovery and Enzyme Engineering (GDEE) pipeline. The enzymes were ranked based on their binding affinity score, and databases and literature were reviewed to finalize the selection of gene sequences. For the *in vivo* implementation **(B)**, various host strains and molecular biology designs were tested. The cells were cultivated considering different media formulations, and the target compounds were quantified using Ultra Performance Liquid Chromatography (UPLC) and confirmed by ^1^H-NMR.

In Figure 1, we provide a schematic representation of this comprehensive workflow.

## 2 Materials and methods

### 2.1 Pathway generation and analysis

Different computational frameworks utilizing enumeration – FindPath (Liu et al., 2015) – and retrobiosynthesis search algorithms – BNICE.ch (Hatzimanikatis et al., 2005) and RetroPath2.0 (Delépine et al., 2018) – were employed to generate pathways for producing target aromatic compounds derived from the shikimate pathway, specifically from tyrosine. Since the results obtained using the various algorithms regarding the compounds studied in this paper were similar, the ShikiAtlas Retrotoolbox (https://lcsb-databases.epfl.ch/SearchShiki) was chosen as the platform for analyzing biosynthetic pathways for L-DOPA and dopamine due to its user-friendly interface. Considering tyrosine as the starting compound, the maximum number of reaction steps was set to 30, and the minimum conservation atom ratio (CAR) was set to 0.34. The pathways were ranked according to the length and the average CAR. ShikiAtlas Retrotoolbox is linked with the computational method and online tool BridgIT (Hadadi et al., 2019), which allows to search and attribute an EC number for each biotransformation constituting the enumerated pathways. The tool Selenzyme (http://selenzyme.synbiochem.co.uk/) (Carbonell et al., 2018) was also used for the same purpose to complement the information obtained from BridgIT. Both tools also provide a first indication of appropriate gene sequences for the desired transformations.

### 2.2 Gene selection: gene discovery and enzyme engineering (GDEE) pipeline

The *in silico* search for enzymes capable of catalyzing the production of L-DOPA, dopamine and tyramine was conducted by an in-house structure-based automated pipeline, called Gene Discovery and Enzyme Engineering (GDEE). Briefly, this pipeline uses an existing 3D structure of a given enzyme, or a predicted 3D model in case the structure does not exist, in order to infer the binding affinity of a given ligand (substrate, intermediate or product), representing the reaction we aim to probe or optimize. As a necessary simplification in this high-throughput approach, binding is considered a proxy for catalytic efficiency. The platform can perform enzyme optimization, where selected positions of a given active site can be mutated (up to hundreds of thousands of enzyme variants) to optimize the enzyme for a transformation, or gene discovery, where natural enzymes are scanned in order to find the ones most prone to catalyze the transformation.

In this work, the gene discovery pipeline was employed. We used the 3D structure of an enzyme, homologous to the candidate enzymes, that is known to catalyze the target or a similar reaction as a starting template to model all enzymes (sequences) that we want to test. The list of candidate enzymes to perform the target reaction comes from a BLASTp search, against the Swiss-Prot database, of all enzyme sequences that are at least 20% identical (minimum coverage of 80%) to the template. In this case, 15 homology-based models are built with Modeller (Šali and Blundell, 1993) for each sequence found in the previous step. To rank all candidate enzymes based on their ability to perform the desired reaction, the five best models (as ranked by the objective function of Modeller) of each candidate enzyme are used to dock a ligand, using AutoDock Vina (Trott and Olson, 2010; Eberhardt et al., 2021), that represents the reaction limiting step. To ensure that ligands exhibit catalytically relevant orientations, distance constraints between atoms from the ligand and the candidate enzyme were used to filter the docking results. After the filtering step, the computed binding affinity is used as a proxy for catalytic efficiency and, thus, utilized to rank the candidate enzymes.

In the case of the reaction catalyzed by Tyrosinase, a compound derived from tyrosine, with another deprotonated oxygen attached to the ring (to mimic a reaction intermediate) was used in the docking calculations to focus the results on the ability of the candidate enzymes to add another hydroxyl group to the phenol ring in an *ortho* position. The box size for docking with AutoDock Vina was set to 18 Å × 15 Å × 16 Å and the exhaustiveness to 100. Docking results were filtered to only allow conformations where the deprotonated oxygen of the phenol is at a distance lower or equal than 3.3 Å from the copper ions present in the active site, and that the *ortho* carbons from the L-tyrosine substrate are at a distance higher than 6.7 Å from the alpha carbon of the E223 residue.

For the DDC reaction, L-DOPA was used as the ligand in the docking calculations to select enzymes that could accommodate the substrate with its amino group in contact with the carbon C4′ of the LLP residue Lys319 linked to the pyridoxal phosphate (PLP) coenzyme in its ζ-amino group, which is crucial for the mechanism of these enzymes. The box size was set to 13 Å × 13 Å × 13 Å and the exhaustiveness to 200. Docking results were filtered to only allow conformations where the nitrogen atom of the L-DOPA substrate was not more than 4 Å away from the C4′ of LLP, and that the C14 of L-DOPA is at a distance lower or equal than 7.5 Å from the alpha carbon of the K295 residue, to guarantee that the aromatic ring was buried in the active site and that the acid group was exposed to the solvent.

For the tyrosine decarboxylase, L-tyrosine was used as the ligand in the docking calculations to find enzymes that could accommodate the substrate with its amino group in contact with the oxygen from the carbonyl functional group of the PLP coenzyme, which is pivotal in the mechanism of these enzymes. The box size was set to 19 Å × 20 Å × 20 Å and the exhaustiveness to 200. Docking results were filtered to only allow conformations where the nitrogen atom of L-tyrosine was not more than 4 Å away from the oxygen of the carbonyl group of PLP, and that the hydroxyl group of L-tyrosine is at a distance lower or equal than 4.2 Å from the alpha carbon of the N100 residue, to ensure that the aromatic ring was buried into the active site and the acid group was exposed to the solvent.

### 2.3 Design of DNA parts

#### 2.3.1 L-DOPA

The gene *tyr* from *Ralstonia solanacearum* (GenBank accession number: AL646052) and respective mutant sequence tyr* (Y119F V153A D137Y L330V) encoding the candidate enzymes to convert L-tyrosine into L-DOPA were cloned in the same fashion into the second multiple cloning site (MCS) of the pETDuet-1 (Novagen) plasmid. The mutant sequence was created by site-directed mutagenesis with the appropriate primers (Supplementary Table S1) containing the desired mutations using the wild-type construct as template.

#### 2.3.2 Dopamine

For the known pathway, the gene encoding L-DOPA decarboxylase (*ddc*) from *Pseudomonas putida* KT2440 (GenBank: BK006920.1) was cloned in the first MCS of the previously constructed plasmids for L-DOPA production.

Regarding the novel pathway for dopamine, the gene encoding the enzyme that catalyzes the first step–tyrosine decarboxylase (*tdc*) from *Levilactobacillus brevis* (GenBank accession number: WP_011668784.1) or the mutant sequence S587A (tdc*) – were cloned in the first MCS of pETDuet-1. Both selected *ppo* genes (one from *Agaricus bisporus* and another from *Mucuna pruriens*; GenBank Accession numbers: AJ223816 and MK140603, respectively) were individually introduced in the second MCS.

### 2.4 Molecular biology protocols

Codon-optimized genes for expression in *Escherichia coli* and primers were purchased from IDT (Iowa, USA). The genes used in this study were amplified by polymerase chain reaction (PCR) using Phusion High-Fidelity DNA Polymerase (Thermo Scientific, Waltham, United States) in a LifeECO Thermal Cycler (Bioer Technology, Zhejiang, China). The commercial plasmid pETDuet-1 from the Novagen pET System (Merck Millipore, Massachusetts, United States) was used to clone heterologous genes. DNA fragments were purified using the DNA Clean and Concentrator DNA Kit (Zymo Research, Irvine, United States). Plasmids were extracted using the ZR Plasmid Miniprep-Classic Kit (Zymo Research).

#### 2.4.1 Assembly of DNA constructs

The assembly of DNA constructs was performed by FastCloning (Li et al., 2011), and compatible primers were designed using the NEBuilder^®^ v2.7.1 Assembly Tool (New England Biolabs, NEB). The resulting assembled parts were transformed by heat-shock into chemically competent *E. coli* DH5α cells (NEB). The size of the final sequence of the DNA constructs was validated by colony PCR using DreamTaq polymerase (Thermo Scientific) and further confirmed by Sanger sequencing (StabVida, Lisbon, Portugal). Cloning was performed in accordance with the manufacturer’s instructions.

#### 2.4.2 Site-directed mutagenesis by PCR

Gene sequences for mutant enzymes were obtained with site-directed mutagenesis by performing two PCR steps using Phusion polymerase (Thermo Scientific) and primers that included the desired mutation. All primers were designed using the QuickChange^®^ Primer Design tool from Agilent (Novoradovsky et al., 2005).

Site-directed mutagenesis was performed in two PCR steps following a protocol adapted from (Reikofski and Tao, 1992) and using the primers listed in Supplementary Table S1 for each target mutation. In the first step, two single-primer reactions were performed following the manufacturer’s instructions but limiting the cycles of amplification to 5. In the second step, 25 μL of each completed reaction was mixed with 1 U of Phusion polymerase in a new PCR tube and amplified for 30 cycles. Finally, the PCR mix was digested with 1 μL of DpnI (ThermoScientific) for 3 h at 37°C, purified and transformed into DH5α chemically competent cells. The constructs containing the desired mutations were identified by Sanger sequencing (StabVida) and used as template for the following round in the case of multiple mutations.

#### 2.4.3 Replacement of promoters

The replacement of T7 promoters in the pETDuet-1 plasmid by *trc* promoters was performed using FastCloning (Li et al., 2011). Chimeric primers were designed to linearize the plasmid excluding the T7 promoter and *lacO* operator. These primers also contained the new *trc*+*lacO* region, with an overlap of 20 bp between each end, allowing further recircularization *in vitro* (Supplementary Table S1). The final amplified fragments were digested with *Dpn*I (Thermo Scientific) for 3 h, purified and transformed into DH5α chemically competent cells. The constructs with the new promoter were identified by Sanger sequencing.

The success of the plasmid constructions was confirmed by sequencing the regions of interest with the appropriate primers. All plasmids used or constructed in this study, as well as the respective major features are described in Table 1.

**TABLE 1:** List of plasmids used or developed in this study.

*Name*	*Feature*	*Source*	*Pathway*
pCas	*repA101(Ts) kan P*_*cas*_ *-cas9 P*_*araB*_ *-Red lacI* ^*q*^ *P*_*trc*_ *-sgRNA-pMB1*	Addgene Jiang et al. (2015)	-
pTargetF	Constitutive expression of sgRNA without donor editing template DNA	Addgene Jiang et al. (2015)	-
pETDuet-1	ColE1(pBR322) *ori*, *lacI*, double T7lac, *Amp* ^*R*^	Novagen	-
pETDuet_P*trcs*	pETDuet-1 with *trc* promoters instead of T7 in both MCS	This study	-
pETDuet_*tyr*	pETDuet-1 carrying *tyr* gene from *Ralstonia solanacearum*	This study	LD
pETDuet_tyr*	pETDuet-1 carrying *tyr* gene from *R. solanacearum* encoding the mutations Y119F_V153A_D137Y_L330V	This study	LD
pETDuet_P*trc2*_*tyr*	pETDuet_*tyr* with the second T7 promoter exchanged by the *trc* promoter	This study	LD
pETDuet_P*trc2*_tyr*	pETDuet_tyr* with the second T7 promoter exchanged by the *trc* promoter	This study	LD
pETDuet *_ddc*	pETDuet-1 carrying *ddc* gene from *Pseudomonas putida*	This study	DPM
pETDuet_*tyr_ddc*	pETDuet_*tyr* carrying *ddc* gene from *P. putida*	This study	DPM
pETDuet_tyr*_ddc	pETDuet_tyr* carrying *ddc* gene from *P. putida*	This study	DPM
pETDuet_P*trcs*_*tyr_ddc*	pETDuet_P*trcs* carrying *tyr* from *R. solanacearum* and *ddc* from *P. putida*	This study	DPM
pETDuet_P*trcs*_tyr*_ddc	pETDuet_P*trcs* carrying tyr* (mutations Y119F_V153A_D137Y_L330V) from *R. solanacearum* and *ddc* from *P. putida*	This study	DPM
pETDuet*_tdc*	pETDuet-1 carrying *tdc* from *Levilactobacillus brevis*	This study	DPA
pETDuet_tdc*	pETDuet-1 carrying *tdc* from *L. brevis* encoding the mutation S587A	This study	DPA
pETDuet_P*trcs_tdc*	pETDuet_P*trcs* carrying *tdc* from *L. brevis*	This study	DPA
pETDuet*_tdc_ppoAB*	pETDuet*_tdc* carrying *ppoAB* from *Agaricus bisporus*	This study	DPA
pETDuet_P*trcs _tdc_ppoAB*	pETDuet_P*trcs* carrying *tdc* from *L. brevis and ppoAB* from *A. bisporus*	This study	DPA
pETDuet*_tdc_ppoMP*	pETDuet*_tdc* carrying *ppoMP* from *Mucuna pruriens*	This study	DPA
pETDuet_P*trcs_tdc_ppoMP*	pETDuet_P*trcs* carrying *tdc* from *L. brevis* and *ppoMP* from *M. pruriens*	This study	DPA
pETDuet_tdc*_ppoMP	pETDuet-1 carrying *tdc* from *L. brevis* encoding the mutation S587A and *ppoMP* from *M. pruriens*	This study	DPA
pETDuet*_ppoMP*	pETDuet-1 carrying *ppoMP* from *Mucuna pruriens*	This study	DPA

MCS, Multiple Cloning Site; LD, L-DOPA; DPM, Dopamine known pathway; DPA, Dopamine novel pathway.

### 2.5 Bacterial strains

After confirmation of the constructs by sequencing, the plasmids were transformed into *E. coli* BL21 (DE3), JM109 (DE3) and K12 MG1655 (DE3) strains for gene expression under control of the T7 or *trc* promotors. All strains used or engineered in this study are summarized in Table 2.

**TABLE 2:** List of strains used in this study.

Strain	Host	Relevant genotype	Source
*Escherichia coli* DH5α	-	*fhuA2Δ(argF-lacZ)U169 phoA glnV44 Φ80Δ(lacZ)M15 gyrA96 recA1 relA1 endA1 thi-1 hsdR17*	NEB
*Escherichia coli* BL21 (DE3)	-	F¯ ompT gal dcm lon hsdSB(rB- mB-) λ(DE3 *lacI lacUV5-T7 gene 1 ind1 sam7 nin5])	Nzytech
*Escherichia coli* JM109 (DE3)	-	*endA1, recA1, gyrA96, thi, hsdR17 (rk–, mk+), relA1, supE44, λ–, Δ(lac-proAB), [F′, traD36, proAB, lacIqZΔM15], lDE3*	Promega
TYR1	*Escherichia coli* BL21 (DE3)	*ΔpheLA ΔtyrR*	This study
TYR2	*Escherichia coli* JM109 (DE3)	*ΔpheLA ΔtyrR*	This study
LD1	*E. coli* BL21 (DE3)	pETDuet_*tyr*	This study
LD2	*E. coli* JM109 (DE3)	pETDuet_*tyr*	This study
LD3	*E. coli* BL21 (DE3)	pETDuet_tyr*	This study
LD4	*E. coli* JM109 (DE3)	pETDuet_tyr*	This study
LD5	*E. coli* MG1655	pETDuet_*tyr*	This study
LD6	*E. coli* MG1655	pETDuet_tyr*	This study
LD7	*E. coli* BL21 (DE3)	pETDuet_P*trc2*_tyr*	This study
LD8	*E. coli* JM109 (DE3)	pETDuet_P*trc2*_tyr*	This study
LD9	*TYR1*	pETDuet_tyr*	This study
LD10	*TYR2*	pETDuet_tyr*	This study
LD11	*TYR1*	pETDuet_P*trc2*_tyr*	This study
LD12	*TYR2*	pETDuet_P*trc2*_tyr*	This study
DPM1	*E. coli* BL21 (DE3)	pETDuet_*tyr_ddc*	This study
DPM2	*E. coli* JM109 (DE3)	pETDuet_*tyr_ddc*	This study
DPM3	*E. coli* BL21 (DE3)	pETDuet_tyr*_ddc	This study
DPM4	*E. coli* JM109 (DE3)	pETDuet_tyr*_ddc	This study
DPM5	*E. coli* BL21 (DE3)	pETDuet*_ddc*	This study
DPM6	*E. coli* JM109 (DE3)	pETDuet*_ddc*	This study
DPM7	*E. coli* K12 MG1655	pETDuet_*tyr_ddc*	This study
DPM8	*E. coli* K12 MG1655	pETDuet_tyr*_ddc	This study
DPM9	*E. coli* BL21 (DE3)	pETDuet_P*trcs*_*tyr_ddc*	This study
DPM10	*E. coli* JM109 (DE3)	pETDuet_P*trcs*_*tyr*_*ddc*	This study
DPM11	*E. coli* BL21 (DE3)	pETDuet_P*trcs*_tyr*_ddc	This study
DPM12	*E. coli* JM109 (DE3)	pETDuet_P*trcs*_tyr*_ddc	This study
DPM13	*TYR1*	pETDuet_tyr*_ddc	This study
DPM14	*TYR2*	pETDuet_tyr*_ddc	This study
DPM15	*TYR1*	pETDuet_P*trcs*_tyr*_ddc	This study
DPM16	*TYR2*	pETDuet_P*trcs*_tyr*_ddc	This study
DPA1	*E. coli* BL21 (DE3)	pETDuet*_tdc_ppoAB*	This study
DPA2	*E. coli* BL21 (DE3)	pETDuet*_tdc_ppoMP*	This study
DPA3	*E. coli* JM109 (DE3)	pETDuet*_tdc_ppoAB*	This study
DPA4	*E. coli* JM109 (DE3)	pETDuet*_tdc_ppoMP*	This study
DPA5	*E. coli* BL21 (DE3)	pETDuet_P*trcs_tdc_ppoAB*	This study
DPA6	*E. coli* BL21 (DE3)	pETDuet_P*trcs_tdc_ppoMP*	This study
DPA7	*E. coli* JM109 (DE3)	pETDuet_P*trcs_tdc_ppoAB*	This study
DPA8	*E. coli* JM109 (DE3)	pETDuet_P*trcs_tdc_ppoMP*	This study
DPA9	*E. coli* BL21 (DE3)	pETDuet_tdc*_ppoMP	This study
DPA10	*E. coli* JM109 (DE3)	pETDuet_tdc*_ppoMP	This study
DPA11	*E. coli* BL21 (DE3)	pETDuet_Ptrcs_tdc*_ppoMP	This study
DPA12	*E. coli* JM109 (DE3)	pETDuet_Ptrcs_tdc*_ppoMP	This study
DPA13	*TYR1*	pETDuet_tdc*_ppoMP	This study
DPA14	*TYR2*	pETDuet_tdc*_ppoMP	This study
DPA15	*TYR1*	pETDuet_Ptrcs_tdc*_ppoMP	This study
DPA16	*TYR2*	pETDuet_Ptrcs_tdc*_ppoMP	This study
DPA17	*E. coli* BL21 (DE3)	pETDuet_*tdc*	This study
DPA18	*E. coli* BL21 (DE3)	pETDuet_*ppoMP*	This study

*E. coli* K12 (MG1655 and JM109) and B (BL21) strains were selected for heterologous protein expression, given their widespread use in industrial-scale applications (Marisch et al., 2013). These strains possess distinct metabolic characteristics, particularly in the glyoxylate shunt and acetate metabolism, highlighting the importance of testing different biosynthetic pathways in diverse metabolic environments to identify the most suitable one for a certain compound. Specifically, the JM109 strain, characterized by its *recA^–^* and *endA^–^* traits, was included to enhance plasmid stability (Yang et al., 2022). Additionally, BL21 cells were used in our study considering their efficient plasmid and soluble protein expression (Marisch et al., 2013). This efficiency is attributed to the lack of Lon and OmpT proteases in BL21 cells (Marisch et al., 2013).

The strains used in this study were obtained by transforming electrocompetent strains with the indicated plasmids by electroporation using 0.1 cm-gap electroporation cuvettes at a voltage of 1.8 kV. Electrocompetent cells were prepared using the protocol developed by Dower et al. (1988). Positive transformants were isolated on Luria-Bertani (LB) agar plates, containing ampicillin and incubated overnight at 37°C. To confirm the success of the transformation, selected transformant colonies were cultivated overnight in LB liquid medium with antibiotic. Subsequently, plasmids were extracted and digested with appropriate restriction enzymes. The correct fragment lengths were confirmed by agarose gel electrophoresis.

LB medium contained 10 g/L peptone, 5 g/L yeast extract and 5 g/L NaCl. For preparation of solid media 15 g/L agar was added. The medium was supplemented with ampicillin at a concentration of 50 μg/mL, when required.

#### 2.5.1 Gene knockouts

Gene knockouts (Δ*pheLA* and Δ*tyrR*) were introduced in *E. coli* BL21 (DE3) and JM109 (DE3) to increase the metabolic flux towards tyrosine. TyrR is involved in the transcriptional repression of the biosynthesis and transport of Aromatic Amino Acids (AAA) (Das et al., 2018). Since prephenate is a precursor for both tyrosine and phenylalanine, to prevent the loss of prephenate in the production of phenylalanine, the gene encoding prephenate dehydratase and its leader peptide (*pheLA*) were deleted, increasing the flux of prephenate towards tyrosine (Wei et al., 2016). The gene deletion was performed using *Streptococcus* pyogenes type II CRISPR-Cas9 following the protocol developed by Jiang et al. (2015). The selection of the 20-bp region complementary to the targeting region (N20) of the guide RNA (gRNA) to each gene was performed with the CRISPR gRNA Design Tool in Benchling [“Benchling” (Biology software). 2021]. The primers used to perform the gene knockouts are listed in Supplementary Table S1.

### 2.6 Shake flask experiments

L-DOPA and dopamine production experiments were performed in M9 medium, containing (per liter): 1 g of NH_4_Cl, 0.5 g of NaCl, 3 g of KH_2_PO_4_, 12.8 g of Na_2_HPO_4_ · 7H_2_O, 11 mg of CaCl_2_ and 240 mg of MgSO_4_. The pH of this medium was adjusted to 7.2 ± 0.2 at 25°C with NaOH.

A single colony was picked from LB plates and inoculated in 10 mL of liquid LB medium with the appropriate antibiotic. The pre-cultures were grown aerobically on a rotary shaker at 37°C and 180 rpm, overnight. An appropriate volume of cells was harvested from the pre-culture by centrifugation (10 min at 3,000×*ɡ*), washed and then transferred to 250-mL shake flasks with 50 mL of M9 medium supplemented with 10 g/L of glucose and the appropriate antibiotic concentration, yielding an initial optical density at *λ* 600 nm (OD600) of 0.1. These cultures were also cultivated on a rotary shaker at 180 rpm at 37°C. The expression of heterologous genes was induced with 0.1–0.5 mM IPTG at an OD600 of 0.4–0.5. At induction point, the culture was supplemented with 1 g/L of tyrosine. Considering the cofactor requirements of the enzymes, 50 μM vitamin B6 was supplemented in dopamine production experiments, while 0.45 g/L L-ascorbic acid was added to the medium in the L-DOPA and dopamine known pathway experiments. Lastly, 25 μM of CuSO_4_ was only supplemented when expressing the novel pathway for dopamine. After IPTG induction, the temperature was reduced to 28°C and the stirring speed to 150 rpm, and cells were cultivated for 144 h. Samples were taken every 24 h for OD600 measurements and UPLC analysis of the supernatant. All experiments were performed with three biological replicates. All titers are expressed as the mean ± standard deviation.

### 2.7 Quantitative analysis

The target compounds were quantified by Ultra Performance Liquid Chromatography (UPLC) using a Waters (Massachusetts, United States) ACQUITY UPLC system equipped with a Photodiode Array (PDA) detector and a Symmetry C18 reverse phase column (5 μm × 4.6 mm × 250 mm) of pore size 100  Å. For the analysis, 1 mL of cell culture was collected and centrifuged at maximum speed for 5 min, then the cell-free supernatant was collected and used for further analysis. 50 μL of sample were injected using a gradient solution with two solvents: (A) ultra-pure water with 1% (v/v) of trifluoroacetic acid (TFA) and (B) a buffer containing 70% of methanol and 30% of acetonitrile with 0.05% (v/v) of TFA. The fraction of B increased linearly from 5% to 70% from 3 to 10 min after injection. Then the fraction of B decreased back to 5% between 10 and 12 min, and remained constant until 20 min. The column temperature was maintained at 35°C. L-tyrosine, tyramine, L-DOPA and dopamine were analyzed at 220 nm.

For NMR analysis, 1 mL of cell culture was collected and centrifuged at maximum speed for 5 min. Subsequently, 540 µL of the cell-free supernatant were transferred to a 5 mm NMR tube with subsequent addition of 60 μL of D_2_O with 3.2 mM of 3-(trimethylsilyl)propionic-2,2,3,3-d_4_ acid (TSP), an internal standard that was used as chemical shift reference and concentration standard.

^1^H-NMR spectra were obtained at 25°C in an Ultrashield Avance 500 Plus spectrometer (Bruker) equipped with a TCI-Z probe, using a *noesypr1d* pulse program (time domain 48K, number of scans 64, relaxation time 1 s, mixing time 0.1 s, sweep width 12 ppm). Spectra were acquired and processed using TopSpin 4.1 software (Bruker) and the compound quantification was done on Chenomx NMR Suite 8.11.

## 3 Results

**FIGURE 2:**
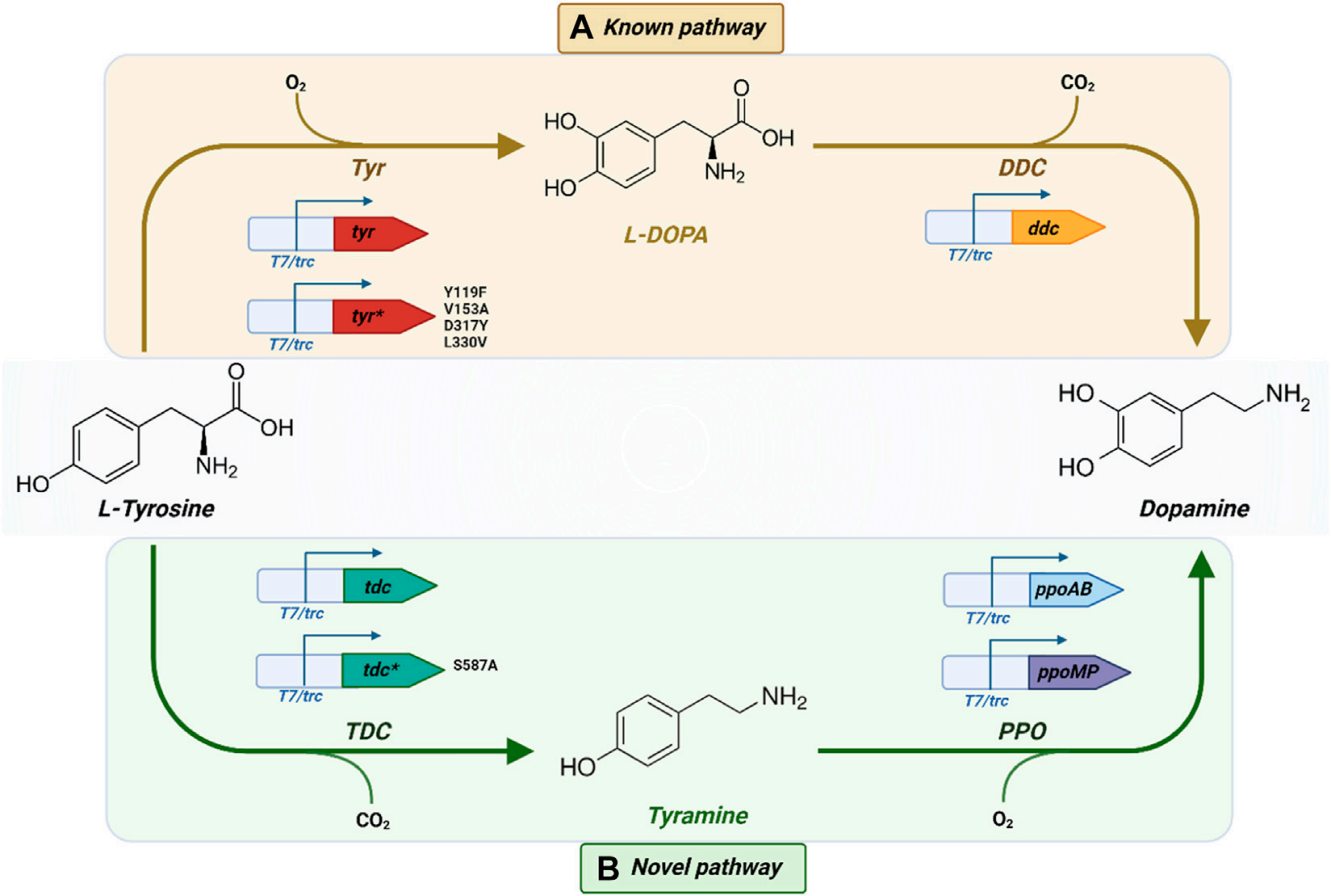
**(A)** Known and **(B)** Novel biosynthetic pathways to produce L-DOPA and dopamine from L-tyrosine including the different molecular biology designs and enzyme variants. *: mutant sequence. *Created* with BioRender.com.

Utilizing the pipeline depicted in Figure 1, different pathways to produce L-DOPA and dopamine were selected, as illustrated in Figure 2. A pathway composed of a single catalytic step was selected for L-DOPA. In the case of dopamine, two different pathways were chosen for *in vivo* implementation: one using L-DOPA as the intermediate compound (Figure 2A) and an alternative one using tyramine instead (Figure 2B), which, to the best of our knowledge, has not been reported yet. In the following sections, we will provide a detailed analysis of the pathway design and gene candidate selection.

### 3.1 3,4-Dihydroxy-L-phenylalanine (L-DOPA)

**FIGURE 3:**
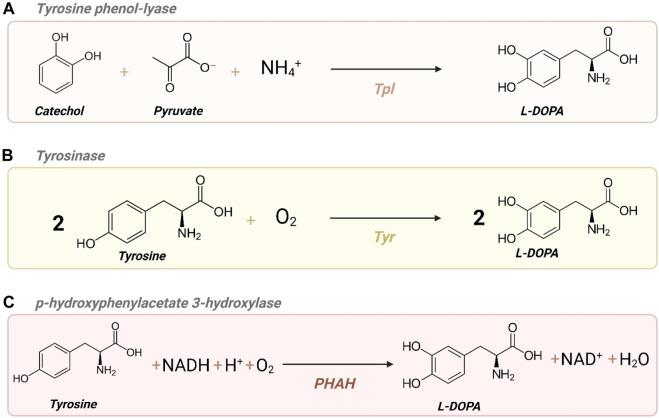
Different enzyme activities to yield L-DOPA. **(A)** *Tpl*: tyrosine phenol-lyase; **(B)** *Tyr*: tyrosinase; **(C)** *PHAH*: *p*-hydroxyphenylacetate 3-hydroxylase. *Created with* BioRender.com.

L-DOPA – also known as levodopa and L-3,4-dihydroxyphenylalanine – is an alpha amino acid and the precursor of the neurotransmitter dopamine. Essentially, three different enzymatic mechanisms can yield L-DOPA as illustrated in Figure 3: tyrosine phenol-lyase (Tpl) converts catechol, pyruvate, and ammonia into L-DOPA (Figure 3A). Additionally, L-DOPA can be produced from L-tyrosine by two different types of enzymes: tyrosinase (Tyr) (Figure 3B) and *p*-hydroxyphenylacetate 3-hydroxylase (PHAH) (Figure 3C; Min et al., 2015).

The initial biotechnological efforts to produce L-DOPA involved leveraging native enzyme activities within organisms. Ajinomoto Co. Ltd pioneered a fermentation process in 1993 using *Erwinia herbicola*, exploiting its native Tpl activity to produce L-DOPA from catechol (Iizumi et al., 1991; Figure 3A). Subsequent research investigated expressing the *tpl* gene from *E. herbicola* in established industrial microorganisms like *E. coli* (Foor et al., 1993). Alternative approaches have since emerged, such as exploring the PHAH mechanism depicted in Figure 3C by expressing *hpaBC* in *E. coli* (Fordjour et al., 2019). Nakagawa et al. developed a bacterial platform to produce plant alkaloids, including L-DOPA and dopamine, by exploring the expression of different tyrosinases (Tyr) (Figure 3B) in *E. coli*, achieving the highest titers with Tyr from *R. solanacearum* (Nakagawa et al., 2011).

#### 3.1.1 Pathway design

Considering the abundance of enzymes catalyzing tyrosine into L-DOPA and respective gene candidates available, we have implemented a computational workflow to support the search for the most efficient pathways. On one hand, this workflow allows us to leverage the accumulated knowledge reported in databases, aiming to select the most suitable pathways and their respective gene sequences for expression in *E. coli*. On the other hand, it also has the advantage of potentially unveiling new routes, either by incorporating orphan reactions (i.e., reactions without enzyme assignment) (Hadadi et al., 2019) or by enumerating new combinations of catalytic steps that are different from those already described in the literature. Lastly, by focusing solely on the best gene candidates, it is easier to conduct a more comprehensive analysis of the studies related to the selected genes, even for different purposes.

As anticipated, given the simplicity of this particular conversion, the highest ranked pathways listed in ShikiAtlas consisted of already known single catalytic steps originating from tyrosine. When considering pathways that employed orphan reactions, they ranked much lower due to the involvement of multiple steps and complex intermediates. The single-reaction pathways featured two distinct mechanisms: one mediated by tyrosinase (Figure 3B) and the other by PHAH (Figure 3C). To identify the most promising candidate enzyme for each enzymatic mechanism, BridgIT and Selenzyme were employed to assign the respective EC numbers and suggest preliminary gene candidates (Supplementary Table S2). The attributed EC numbers with a reaction similarity score equal to 1 for tyrosinase were 1.10.3.1 and 1.14.18.1, while EC 1.14.14 and 1.14.16.2 were assigned for PHAH (Supplementary Table S2). Notably, in the case of PHAH, the *E. coli* sequence (*hpaBC*) received a significantly higher score compared to other enzymes (Supplementary Table S3). Due to the limited sequence diversity available for this mechanism, the need for biochemical reducing power, and the prior extensive exploration of this enzyme and its mutant sequences, we chose to focus our efforts on the tyrosinase mechanism and test PHAH only in control strains. Conversely, for the tyrosinase reaction, enzyme selection tools proposed multiple enzymes with identical scores. However, this list failed to include successfully reported sequences for catalyzing the conversion of tyrosine into L-DOPA. Therefore, we implemented the GDEE pipeline to assist in gene candidate selection by conducting a more in-depth analysis of the reaction mechanism to choose the most likely enzyme with the highest activity.

#### 3.1.2 Gene sequence selection

Considering the EC number attributed by BridgIT and Selenzyme and the data retrieved from literature and databases, a tyrosinase from *R*. *solanacearum* was used as the template for the *in silico* search of other sequences, due to its validated expression and high activity in *E. coli* (Hernández-Romero et al., 2006). A homology-based model was created for this enzyme with Modeller, using the X-ray structure of the holo-form of a polyphenol oxidase from *Solanum lycopersicum* (PDB code 6HQI) (Kampatsikas et al., 2019), which has a protein sequence 24% identical to the tyrosinase from *R. solanacearum*. Additionally, we also used in the comparative modelling method a model of tyrosinase from *R. solanacearum* generated using AlphaFold2 (Jumper et al., 2021; Varadi et al., 2022). The list of candidate enzymes was obtained by a BLASTp search against the SwissProt database using the protein sequence of the polyphenol oxidase from *R. solanacearum*.

A total of 24 candidate enzymes were chosen after filtering the BLAST results (the complete list can be found in Supplementary Table S4). These enzymes originate from a wide range of organisms. Interestingly, eukaryotic enzymes exhibited a higher capability for performing the transformation according to the pipeline (the top five enzymes exhibited binding affinities ranging between −6.4 and −6.7 kcal/mol). However, expressing eukaryotic enzymes in *E. coli* can be challenging due to post-translational modifications and solubility issues and, therefore, these results should be evaluated *in vivo* using eukaryotic hosts in future work. Consequently, since it is the top prokaryotic enzyme returned by the pipeline (Table 3), the above-mentioned tyrosinase from *R*. *solanacearum* was selected for *in vivo* implementation. It is worth noting that this enzyme has been reported to exhibit an abnormally high tyrosine hydroxylase:dopa oxidase ratio in comparison to other tyrosinases, which is in accordance with the outcomes obtained through the pipeline (Hernández-Romero et al., 2006). We also found that specific mutations (Y119F; V153A; D317Y; L330V) in this enzyme have been reported to increase the catalytic efficiency towards D-tyrosine, also leading to a 1.4-fold higher L-tyrosine:L-DOPA activity ratio compared to the wild-type (WT) in kinetic experiments (Molloy et al., 2013). This mutant sequence was never tested in a biosynthetic pathway. Therefore, both WT and mutant variants of *tyr* were tested to produce L-DOPA.

**TABLE 3:** Top six enzyme candidates obtained for tyrosinase activity using the gene-discovery pipeline, organized by their binding affinity score (obtained by Autodock Vina), along with their corresponding UniProt ID.

Rank	Uniprot ID	Δ*G (*kcal/mol)	Organism
1	Q9MB14	−6.7	*Ipomoea batatas*
2	Q08296	−6.6	*Solanum lycopersicum*
3	Q08305	−6.4	*Solanum lycopersicum*
4	Q08303	−6.4	*Solanum lycopersicum*
5	Q06355	−6.4	*Solanum tuberosum*
6	Q8Y2J8*	−6.1	*Ralstonia solanacearum*

Enzymes from prokaryotic sources are highlighted. The Uniprot ID of the enzyme used as a template is marked with an asterisk. The complete list can be found in Supplementary Table S4.

#### 3.1.3 *In vivo* results

**FIGURE 4:**
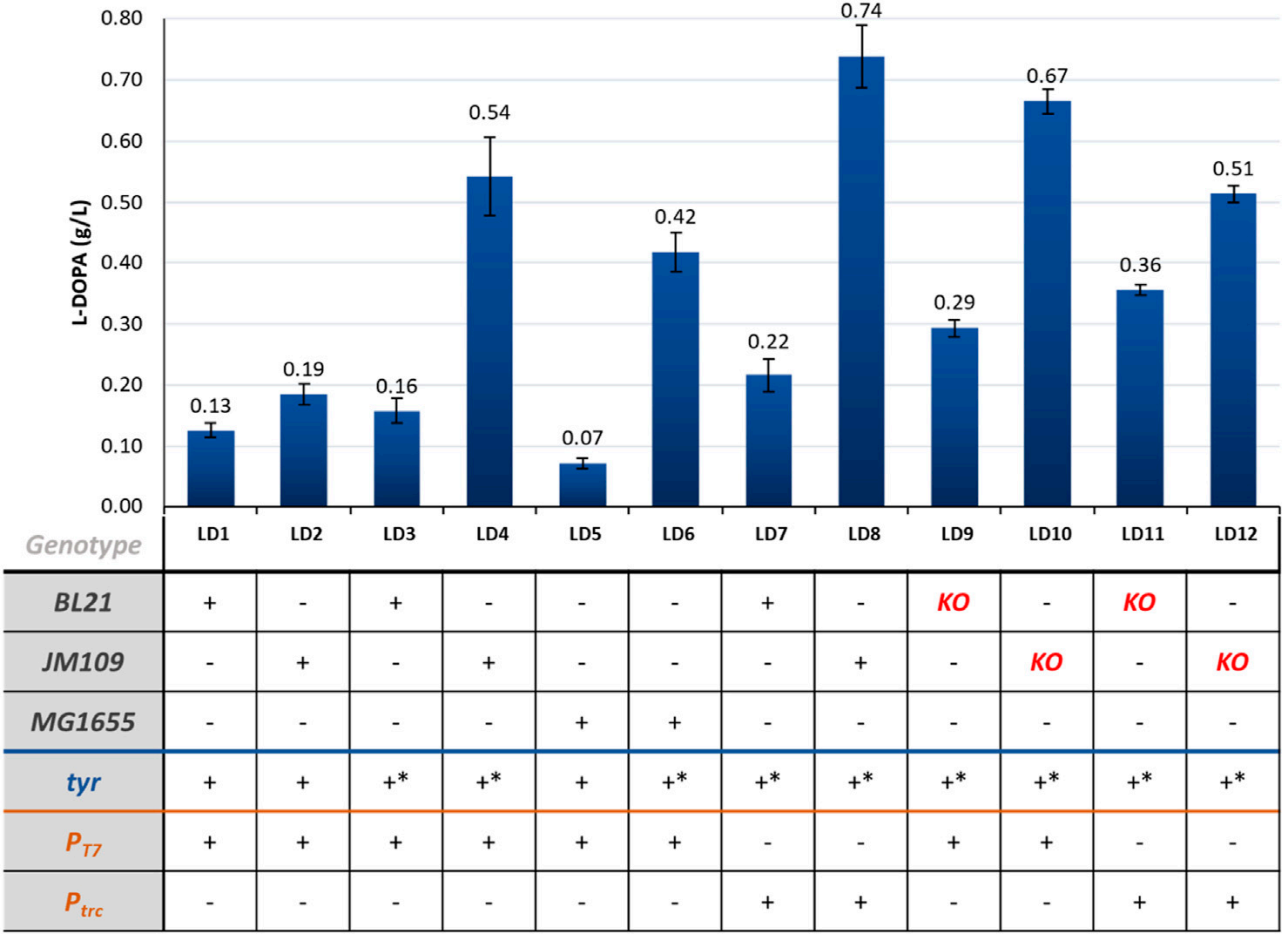
L-DOPA production in the different engineered strains. KO = ΔpheLA ΔtyrR; *- mutant sequence.

The main *in vivo* results are shown in Figure 4. After our computational analysis, we chose tyrosinase as the primary enzyme for converting L-tyrosine into L-DOPA. As mentioned, *hpaBC* from *E. coli* was initially meant to be used in control strains in this study. However, despite testing different conditions and molecular biology designs – including the expression of the two subunits (*hpaB* and *hpaC*) under the control of individual promoters – we were unable to detect any L-DOPA production in strains expressing *hpaBC* (data not shown). SDS-PAGE gels were run to confirm the solubility of the expressed enzymes (Supplementary Figure S1). Unlike the mineral media used in our experiments, previous studies used either complex medium such as Terrific Broth or mineral media supplemented with yeast extract and tryptone (Das et al., 2017; Fordjour et al., 2019). Other reports claim that glycerol supplementation increases the availability of NADH, which is an essential cofactor in this reaction (Fordjour et al., 2019). Hence, it is possible that the absence of specific components or cofactors in our media, or an insufficient NADH pool, could explain the lack of L-DOPA production in our study.

In terms of cells expressing Tyr, introducing four mutations in the *tyr* gene from *R*. *solanacearum* led to a 2.8-fold increase in titer from 0.19 g/L (LD2) to 0.54 g/L (LD4). Although these mutations were previously studied *in vitro*, their implementation for *in vivo* L-DOPA production had not been reported until now. Replacing the T7 promoter in the pETDuet_tyr* plasmid with a weaker promoter (*trc*) improved the L-DOPA titer from 0.54 g/L (LD4) to 0.74 g/L (LD8) in JM109 cells, which was the highest titer achieved. This improvement was also observed in BL21 strains, with an increase from 0.16 to 0.22 g/L (strains LD3 and LD7).

We also observed that L-DOPA production appeared to be more favorable in K-based cells as host strains, especially JM109 strains, which achieved higher titers using the same molecular biology designs. Inactivating the regulatory protein (TyrR) and the genes encoding prephenate dehydratase and its leader peptide (*pheLA*) in the tyrosine competing pathway did not lead to higher L-DOPA titers (strains LD9–12).

The NMR spectra for LD strains validated L-DOPA production in selected strains (Supplementary Figure S2).

### 3.2 Dopamine

Dopamine is a catecholamine neurotransmitter synthesized by dopaminergic neurons mostly in three specific regions of the brain (*substantia nigra*, ventral tegmental area and hypothalamus), acting as a precursor of noradrenaline and adrenaline (Juárez Olguín et al., 2016). Dopamine has innumerous applications in health including the treatment of hypotension, heart failure and occasionally septic shock (Juárez Olguín et al., 2016). The lack of comprehensive reports on dopamine production in the literature highlights the need for our computational workflow. Previous studies have achieved moderate dopamine yields, such as 27 mg/L in LB broth by expressing *hpaBC* (to yield L-DOPA) in conjunction with an engineered dopa decarboxylase (*ddc*) from pig kidney (Das et al.). This enzyme requires iron supplementation and was modified to facilitate soluble expression in *E. coli* (Das et al., 2017).

In another study with the primary aim of producing reticuline, the authors developed a dopamine-producing pathway that was able to produce 1.05 g/L of dopamine in a tyrosine chassis strain accumulating 4.37 g/L of tyrosine. This achievement resulted from combining the expression of the tyrosinase from *R. solanacearum* with the *ddc* gene from *P*. *putida* (Nakagawa et al., 2011).

We employed ShikiAtlas to analyze pathways for producing dopamine from tyrosine. After filtering and ranking the results, we selected two pathways for implementation: the known pathway utilizing L-DOPA as the intermediate compound (Figure 2A) and a novel pathway comprising two steps, with tyramine serving as the intermediate compound (Figure 2B).

#### 3.2.1 Dopamine known pathway–design and gene sequence selection

Since we have already validated the conversion of tyrosine into L-DOPA, we have focused here on selecting the best enzyme to catalyze the decarboxylation of L-DOPA into dopamine. Two different EC numbers were attributed by BridgIT and Selenzyme for this reaction as having activity towards L-DOPA yielding dopamine with a reaction similarity score equal to 1: 4.1.1.25 and 4.1.1.28 (Supplementary Table S2). Among those, several aromatic amino acid decarboxylases (AADCs) from various sources were identified Further consideration of other factors, such as phylogenetic distance, UniProt protein evidence and sequence conservation, led to the identification of top-ranked enzymes (Supplementary Table S3) originated from nematode or plants, potentially presenting possible solubility issues when expressed in *E. coli,* while the prokaryotic sequences presented similar scores among themselves, complicating the selection process. To address this concern, we utilized our in-house GDEE pipeline to identify gene candidates derived from prokaryotic organisms that potentially exhibit higher activity towards dopamine.

In the tool, the DOPA decarboxylase from *P. putida* was chosen as the template, since it was described in the literature as a promising candidate to carry out this reaction (Koyanagi et al., 2012). A homology-based model was created for this enzyme with Modeller using the X-ray structure of the aromatic-L-amino-acid decarboxylase from *Catharanthus roseus* (PDB code 6EEW) (Torrens-Spence et al., 2020), which has a protein sequence 40% identical to the DOPA decarboxylase from *P. putida* and catalyzes a reaction mechanistically similar to the L-DOPA-dopamine conversion. The list of candidate sequences was obtained by a BLASTp search of the protein sequence of the DOPA decarboxylase from *P. putida* against the SwissProt database. Since the BLAST search resulted in only few enzymes sharing similarity with the target, the coverage on the filter was reduced to 60% and the sequence identity was raised to 40% to compensate.

A total of 64 enzymes were selected after filtering the BLAST results (Supplementary Table S5). Only five candidate enzymes originated from prokaryotic sources. The DOPA decarboxylase from *P*. *putida* (UniProt ID: Q88JU5) and the aspartate 1-decarboxylase from *Aliivibrio fischeri* (UniProt ID: Q5E6F9) were the highest-ranking candidates, although the pipeline was unable to distinguish which one was better (both have a binding affinity of −5.4 kcal/mol), as shown in Table 4. For *in vivo* testing, we selected the *ddc* gene from *P. putida* due to its prior experimental validation in producing dopamine from L-DOPA (Koyanagi et al., 2012), while *panP* from *A. fischeri* has only been validated for converting aspartate into β-alanine.

**TABLE 4:** Gene discovery pipeline top 3 results for the L-DOPA decarboxylase reaction for both eukaryotes and prokaryotes, along with their respective UniProt IDs, organized by their binding affinity score (obtained by Autodock Vina).

Rank	Uniprot ID	ΔG (kcal/mol)	Organism
1	P54769	−7.2	*Papaver somniferum*
2	Q9M0G4	−7.0	*Arabidopsis thaliana*
3	Q05733	−6.7	*Drosophila melanogaster*
…	…	…	…
38	Q88JU5*	−5.4	*Pseudomonas putida*
39	Q5E6F9	−5.4	*Aliivibrio fischeri*
41	A7B1V0	−4.8	*Ruminococcus gnavus*

Enzymes from prokaryotic sources are highlighted. The Uniprot ID of the enzyme used as a template is marked with an asterisk. The complete list can be found in Supplementary Table S5.

#### 3.2.2 Dopamine novel pathway–design and gene sequence selection

Alternative pathways to produce dopamine were analyzed using the ShikiAtlas platform by selecting tyrosine as the starting compound. A promising pathway of the same size as the known pathway (consisting of 2 reactions) was discovered (Figure 2B). In this pathway, tyrosine is initially converted into tyramine, which, in turn, yields dopamine. To the best of our knowledge, this pathway has never been described with this combination of enzymes or implemented *in vivo* and offers an alternative to the traditional pathway.

For this pathway, BridgIT and Selenzyme were utilized to identify candidate genes for each reaction. In the initial step involving the decarboxylation of tyrosine into tyramine, two EC numbers were attributed (EC 4.1.1.25 and EC 4.1.1.28) with a Reaction Similarity Score equal to 1 (see Supplementary Table S2). The first corresponds to tyrosine decarboxylase, while the latter refers to DOPA decarboxylase (which also exhibits affinity to other substrates, including tyrosine). As the first reaction is specific to this particular conversion, we proceeded with this EC number. The top-ranked enzyme sequences originate from extremophiles as shown in Supplementary Table S3. Therefore, the mild cultivation conditions of *E. coli* may be unsuitable for obtaining maximum enzymatic activity when expressing those genes (Kruglikov et al., 2022). By revising the literature and databases, we found that L-tyrosine decarboxylase from *Levilactobacillus brevis* has validated kinetic data for converting L-tyrosine into tyramine (Zhang and Ni, 2014). Consequently, the X-ray structure of this enzyme, identified with the PDB code 5HSJ, served as the template for the GDEE pipeline. A BLASTp search against the SwissProt database was applied, again with the sequence coverage filter reduced to 60% (and the protein sequence identity raised to 40%). After refining the BLAST outcomes, 3 candidate enzymes were chosen and subsequently ranked utilizing the automated pipeline (Table 5). The L-tyrosine decarboxylase from *Levilactobacillus brevis (tdc),* UniProt ID: J7GQ1, was the best enzyme candidate with a binding affinity of −5.7 kcal/mol. Moreover, this enzyme had already validated soluble expression in *E. coli* (Zhang and Ni, 2014) and therefore was chosen for further experimental validation. A mutant variant of this enzyme (S586A) has demonstrated higher affinity towards tyrosine (Zhu et al., 2016) and, consequently, was also implemented in this study.

**TABLE 5:** Gene discovery pipeline ranked results for the decarboxylation of tyrosine into tyramine with the respective UniProt ID. The Uniprot ID of the enzyme used as a template is marked with an asterisk.

Rank	Uniprot ID	ΔG (kcal/mol)	Organism
1	J7GQ11*	−65.7	*Levilactobacillus brevis*
2	A0A481NV25	−5.6	*Enterococcus faecium*
3	P0DTQ4	−5.2	*Enterococcus faecalis*

The next step in the pathway involves converting tyramine into dopamine. However, this reaction lacks an attributed EC number with Reaction Similarity Score equal to 1 in BridgIT and Selenzyme (Supplementary Table S2), leading to the unavailability of templates for the GDEE platform to search the optimal gene candidate to catalyze this reaction. Through literature search, we have found a reference to cytochrome CYP2D6 capable of catalyzing this reaction (Hiroi et al., 1998). However, expressing functionally active cytochromes in *E. coli*, while possible, is not a trivial task. It typically involves N-terminal sequence modifications, the use of chaperones and specific plasmids (Yun et al., 2006; Pan and Amini, 2011), which may not be favorable in the context of expressing a biosynthetic pathway. Nevertheless, an identifier (ID: MNXR130692) was obtained from MetaNetX via ShikiAtlas, specifically for the biotransformation associated with reaction ID 1569943850 in the internal Laboratory of Computational Systems Biotechnology (LCSB) database. Further exploration of this entry in MetaNetX directed to the external link ID sabiorkR:11664 in the SABIO-RK database. SABIO-RK is a curated database that houses information about biochemical reactions, their kinetic rate equations, parameters, and experimental conditions (Wittig et al., 2018). Within this database, two polyphenol oxidases (PPOs) were identified with validated kinetic parameters for catalyzing this reaction: one sourced from *Agaricus bisporus* (O42713) and another from *Mucuna pruriens* var. *pruriens* (Table 6). Consequently, we decided to experimentally test both gene candidates.

**TABLE 6:** List of enzymes suggested by SABIO-RK (Biochemical Reaction Kinetics Database) for catalyzing the conversion of tyramine into dopamine, along with respective references to experimental data.

Gene	EC number	Protein ID	Reaction	Organism	Reference
*ppoAB*	1.14.18.1	O42713	O_2_ + tyramine = H_2_O + dopamine	*Agaricus bisporus*	Espín et al. (2000)
*ppoMP*	1.14.16.2	-	O_2_ + tyramine = H_2_O + dopamine	*Mucuna pruriens* var. *utilis*	Luthra and Singh (2010b)

Nevertheless, for the latter organism, no gene sequence was available. Bolstered by a literature search, we were able to find a hypothetical gene sequence to test (Saranya et al., 2020). This sequence has been deposited in GenBank with the accession number MK140603. The authors describe this enzyme as a PPO, although they only inferred monophenolase activity with tyrosine. We hypothesized that this enzyme may also exhibit activity towards tyramine and could be the enzyme initially described as tyrosine hydroxylase (TH) (Luthra and Singh, 2010a). In this last study, the authors conducted kinetic experiments and confirmed that the enzyme described as TH accepts tyramine as a substrate.

#### 3.2.3 Dopamine production via the known pathway

**FIGURE 5:**
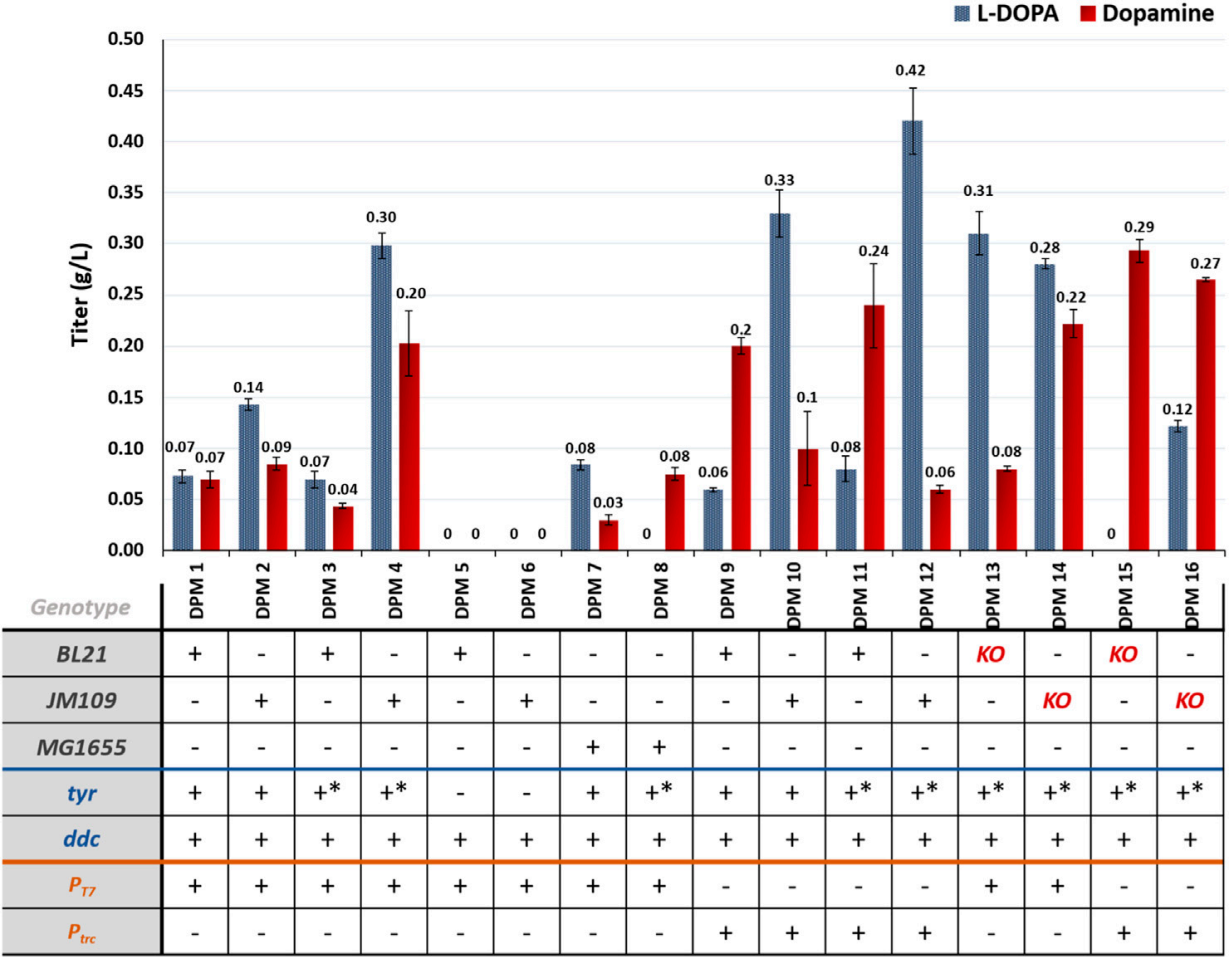
Dopamine and L-DOPA accumulation for the different engineered strains developed for dopamine production via the known pathway. KO = *ΔpheLA ΔtyrR*; *- mutant enzyme.

Results from the *in vivo* implementation of the known dopamine producing pathway can be seen in Figure 5. These results align with the findings from the L-DOPA experiments. Strains expressing *tyr* were able to accumulate L-DOPA, and when coupled with the expression of *ddc*, these strains also produced dopamine. It should be mentioned that almost all strains investigated in this study accumulated both compounds, which indicates potentially that the second step is the limiting one.

When comparing different host strains, it is noteworthy that JM109-based strains expressing the heterologous genes under the control of T7 promoters generally exhibited higher L-DOPA titers compared to BL21-based strains, which is consistent with the L-DOPA results. However, despite the higher L-DOPA titers in JM109-based strains, there were no significant differences in dopamine production among the different strains. This suggests that the second reaction of the pathway, leading to dopamine synthesis, may be favored in BL21-based cells or that there is a kinetic constraint in the second reaction which is not solved by increasing the availability of L-DOPA levels in the ranges observed here.

Interestingly, the use of *trc* promoters led to lower L-DOPA titers in BL21-based strains (DPM9 and DPM11) but higher dopamine titers compared to the JM109 hosts (DPM10 and DPM12). In fact, the maximum dopamine titer achieved in this study was 0.29 g/L in strain DPM15, which was a BL21-based strain. It is worth noting that L-DOPA was not detected in the growth medium of this strain, indicating that the produced L-DOPA was either converted into dopamine or oxidized into dopaquinone. The addition of ascorbic acid to the medium mostly inhibited this spontaneous reaction, although some residual brown coloration was still observed (Supplementary Figure S5).

In terms of pathway designs, the expression of mutant *tyr* and *ddc* under the control of *trc* promoters favored dopamine accumulation in the studied strains, and a similar performance was observed for L-DOPA producing strains.

Regarding the host strains with increased flux towards tyrosine (DPM13-16), no significant differences in dopamine titers were observed compared to their respective wild-type strains. Nevertheless, it is worth noting that the maximum dopamine titer in this study was obtained with strain DPM15 (0.29 g/L), having the genes *pheLA* and *tyrR* deleted, and showing a slight but not statistically significant improvement compared to the wild-type equivalent DPM11 (0.24 g/L).

The NMR spectra for DPM strains validated the L-DOPA and dopamine production (Supplementary Figure S2).

#### 3.2.4 Dopamine production via the novel pathway

**FIGURE 6:**
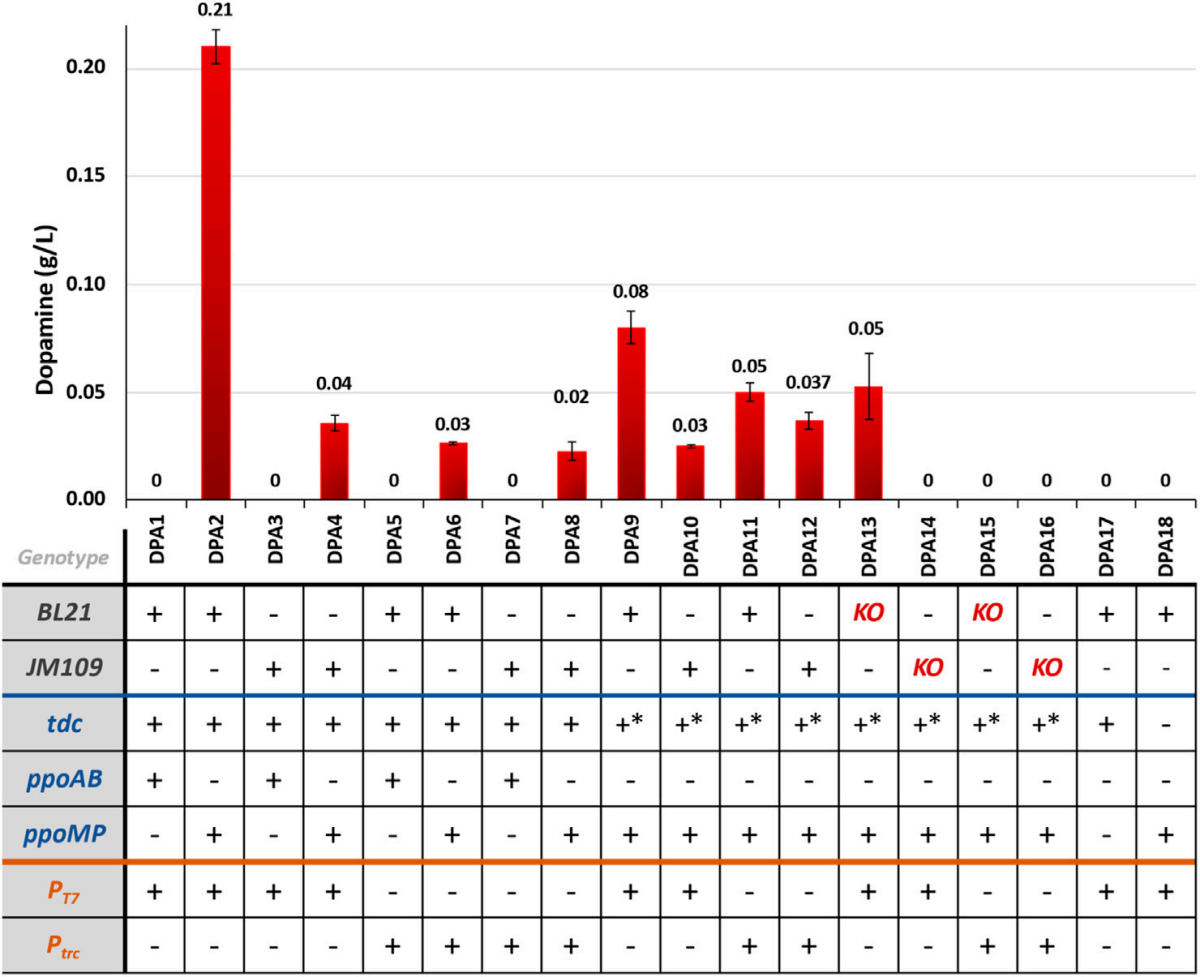
Dopamine production for the different engineered strains for dopamine production via the novel pathway. KO = *ΔpheLA ΔtyrR*; *- mutant sequence.

Among the tested strains for the novel pathway shown in Figure 6, those expressing *ppo* from *M. pruriens* (DPA2, DPA4, DPA6, DPA8) successfully produced dopamine, with titers ranging from 0.02 to 0.21 g/L. In contrast, strains expressing *ppo* from *A. bisporus* (DPA1, DPA3, DPA5, DPA7) did not yield detectable levels of dopamine.

In our experiments, BL21-based strains showed higher dopamine titers compared to JM109-based strains expressing *ppoMP* as depicted in Figure 6. This suggests that the choice of the host strain influenced dopamine production under the tested conditions, which was consistent with the findings observed in the dopamine known pathway. However, the expression of the mutant *tdc*, which was reported to have increased activity towards tyrosine (Zhu et al., 2016), did not result in higher dopamine titers. Notably, tyramine was detected in all samples, surpassing the limit of detection (results not shown), We also tested the individual expression of the two pathway enzymes – *tdc* and *ppoMP* (strains DPA17 and DPA18, respectively). The *tdc*-expressing strains accumulated 0.78 g/L tyramine (Supplementary Figure S4), but dopamine was not detected in either experiment (data not shown), indicating that the expression of both enzymes is required to yield dopamine.

The influence of different promoters on dopamine accumulation remains unclear. In some cases, T7 promoters seemed favorable (DPA2 vs. DPA6), while the use of *trc* promoters resulted in higher dopamine titers when expressing the mutant TDC.

The knock-out strains (*ΔpheLA ΔtyrR*) did not yield higher dopamine titers (strains DPA13-16). In fact, the titers were lower than those achieved with the WT counterparts. Notably, for the knockout strains expressing the genes under the control of *trc* promoters (DPA15 and DPA16), dopamine was not even detected.

The NMR spectra for DPA strains validated the Tyramine and Dopamine production (Supplementary Figure S3).

## 4 Discussion

The selection of suitable biosynthetic pathways to produce target compounds is often a challenging and time-consuming task, given the vast amount of information available in databases and literature. Manual curation alone can be prone to errors and may not fully leverage the accumulated knowledge. The integration of computational tools, such as enumeration/retrosynthesis algorithms and enzyme selection tools, can significantly expedite and enhance the rational design process.

Additionally, the presence of promiscuous enzymes that exhibit activity towards multiple substrates presents a challenge, potentially diminishing pathway efficiency for the desired compound. Even after an enzyme is selected, the task of identifying the optimal gene source to optimize the intended function remains complex. Another difficulty arises from orphan reactions, which lack protein or gene sequences associated, hindering the establishment of complete biosynthetic routes (Wu et al., 2011; Delépine et al., 2018; Hadadi et al., 2019; Sveshnikova et al., 2022b).

To tackle these challenges and enhance the rational design process, we developed a computational workflow that integrates various tools, including enumeration/retrosynthesis algorithms, a toolbox for pathway analysis, enzyme selection tools, and a gene discovery pipeline (Figure 1A). This integrated approach significantly accelerates pathway design, allowing us to leverage accumulated knowledge while exploring novel pathways. This is achieved by enumerating new combinations of enzymatic steps and potentially incorporating orphan reactions.

In this study, we successfully validated the computational workflow for designing biosynthetic pathways to produce tyrosine-derived compounds in *E. coli* – specifically L-DOPA and dopamine – which hold significant applications in health and nutrition. Although prior studies have reported the production of these compounds in *E. coli* (Muñoz et al., 2011; Nakagawa et al., 2011; Wei et al., 2016; Das et al., 2017; Fordjour et al., 2019), our approach employed computational methods to enhance pathway design by searching for the most active enzyme candidate for each step and potentially discover novel pathways. Notably, besides focusing in a known pathway for L-DOPA and dopamine, we were able to find and validate a novel dopamine biosynthetic pathway by expressing a combination of gene sequences that, to the best of our knowledge, has never been reported before.

L-DOPA was selected as a target product since it has become the most widely used medication for treating Parkinson’s disease (PD), as it effectively increases the dopamine levels in the brain (Min et al., 2013). Unlike dopamine, L-DOPA can cross the blood–brain barrier (BBB), where it is converted into dopamine by the action of aromatic L-amino acid decarboxylase. Currently, most of the commercially available L-DOPA is chemically synthetized by Monsanto using the asymmetric synthesis method (Min et al., 2015). The primary drawbacks of converting tyrosine into L-DOPA are the formation of the by-product L-dopaquinone and the consequent requirement for expensive reducing agents (such as ascorbic acid) or electrical reducing power (Min et al., 2015). These challenges, coupled with the increasing demand for L-DOPA in response to the growing elderly population, have prompted the development of biotechnological alternatives. In fact, PD is estimated to affect almost 8.5 million individuals by the World Health Organization (WHO) in 2019 (WHO, 2022). In recent years, alternative methods using enzymes and/or microorganisms have been explored to obtain this dopaminergic drug. For L-DOPA production, our strategy involved implementing a pathway that incorporates a known reaction and introduces a mutant *tyr* enzyme with enhanced activity, which had not been previously tested *in vivo*, to yield L-DOPA (Molloy et al., 2013). This approach resulted in a maximum titer of 0.71 g/L, achieved using the mutant enzyme (Figure 4). The highest reported titer to date was 0.691 g/L when expressing a mutant version of *hpaBC* (PHAH activity) (Figure 2C) in a tyrosine chassis strain (Fordjour et al., 2019). However, while in the referenced study a rich medium was used, we obtained our product titers in mineral media. Robinson et al. have explored the wild-type sequence of the same enzyme we used to yield L-DOPA (*tyr* from *R. solanacearum*), resulting in the production of L-DOPA with a maximum titer of around 20 mg/L (Robinson et al., 2020). Nakagawa et al. developed a bacterial platform to produce plant alkaloids, including L-DOPA as intermediate compound. They explored the expression of different tyrosinases (Figure 3B) in *E. coli*, originating from *Homo sapiens*, *Pholiota nameko* and *Streptomyces castaneoglobisporus* achieving a maximum L-DOPA titer of 293 mg/L using Tyr from *S. castaneoglobisporus*. These authors mentioned a notable 7-fold increase in the final product (reticuline) when substituting the tyrosinase from *S. castaneoglobisporus* with the sequence from *R. solanacearum* (Nakagawa et al., 2011). Direct comparisons between the titers we achieved and those reported in the literature are challenging due to disparities in experimental conditions, such as the use of tyrosine-chassis strains versus direct tyrosine supplementation and the use of complex medium versus mineral media. Nevertheless, the maximum titer we achieved represents a promising milestone, slightly surpassing the maximum titer obtained with the PHAH mechanism. Additionally, this marks the first instance of the mutant tyrosinase being utilized for the production of L-DOPA within a biosynthetic pathway.

Our second target compound, dopamine, is involved in several physiological functions like reward, motivation, learning and memory and its dysfunction can lead to various nervous system diseases. Dopamine imbalances in the brain, either by excess or deficit, have been associated with several conditions including depression, schizophrenia, insomnia, attention deficit hyperactivity disorder, and PD. As mentioned before, since dopamine is unable to cross the BBB, L-DOPA has been used to treat PD by increasing dopamine levels (Min et al., 2013). Nevertheless, dopamine has innumerous applications in health including as a therapeutic agent for acute circulation disorders and cardiovascular stimulation (Juárez Olguín et al., 2016). Recently, it has garnered attention in the field of advanced carbon materials, as dopamine-based N-doped carbon is vital for future carbon electrodes (Dong et al., 2023). With the global dopamine market revenue surpassing $320 million in 2022 and an expected 8.2% annual growth (Dopamine Market Report 2023; Global Edition, Cognitive Market Research), the demand for sustainable processes intensifies.

We explored two distinct biosynthetic pathways for dopamine production: the established pathway reported in the literature (Figure 2A), with the aim of optimizing and implementing the most suitable sequences possible, and a novel pathway (Figure 2B) generated through enumeration and retrobiosynthesis algorithms. The known pathway achieved a maximum titer of 0.29 g/L, while the novel pathway demonstrated equivalent efficacy with a titer of 0.21 g/L Both production strains were cultivated under identical conditions to ensure fair comparison. The comparable efficacy of the novel pathway, achieving 72% of the titer obtained with the known pathway, underscores its potential as a viable alternative for dopamine production Previous reports in the literature state a dopamine titer of 26 mg/L when combining *hpaBC* with an engineered *ddc* from pig kidney (Fordjour et al., 2019) and the accumulation of 1.05 g/L when expressing *tyr* from *R. solanacearum* and *ddc* from *P. putida* (Nakagawa et al., 2011). The authors chose this decarboxylase because it has been identified as a eukaryotic-type aromatic amino acid decarboxylase with a unique substrate specificity that sets it apart from previously characterized prokaryotic AADCs (Koyanagi et al., 2012). In this last work, the authors expressed these enzymes in a tyrosine-chassis strain, which was able to accumulate 4.37 g/L of this aromatic amino acid. This means that the product yield on substrate (g/g) was 24% at maximum, as the cells were grown in a complex medium that also contains tyrosine. The titer achieved with the known pathway in our work corresponds to a 29% conversion yield of tyrosine into dopamine, which represents a slight improvement over what has been reported previously. The novel pathway demonstrated a 21% conversion yield of tyrosine into dopamine. To the best of our knowledge, this marks the first successful implementation of an alternative pathway for dopamine production in *E. coli*. Like the known pathway, this novel route also consists of two catalytic steps. However, it circumvents the use of L-DOPA as the intermediate compound, using tyramine instead. It is worth noting that by the action of tyrosinase, L-DOPA has a propensity to oxidize into *o*-dopaquinone, a structurally similar compound, which then undergoes a series of spontaneous oxidative reactions to yield melanin (Molloy et al., 2013). The initial oxidation step carried out by tyrosinase reduces overall process efficiency by degrading dopamine’s precursor and simultaneously occupying tyrosinase active centers with L-DOPA instead of tyrosine. Ascorbic acid can reduce the oxidation reaction, but it has the drawback of being a costly supplement that can increase overall bioprocess costs. Additionally, it acts as an inhibitor in the conversion of tyrosine into L-DOPA (Min et al., 2013; Nokinsee et al., 2015). Consequently, the enumerated pathway mitigates the issue of L-DOPA degradation into melanin-like products and eliminates the need for ascorbic acid supplementation (Min et al., 2015).

Notably, in this novel dopamine strain, an active form of a polyphenol oxidases (PPO) was employed within the context of a biosynthetic pathway for the first time. PPOs are part of the type III copper family of metalloenzymes. These enzymes are widely distributed and exhibit both monophenolase and diphenolase activities (Panis et al., 2020; Saranya et al., 2023). In our investigation, we focused on the first activity, in which the enzyme transforms tyramine into dopamine. One peculiarity of this class of enzymes lies in the fact that the sequence encodes a latent pro-enzyme containing the catalytically active domain and a C-terminal domain that blocks the entrance to the catalytic pocket, and it must be removed to obtain an active enzyme (Panis and Rompel, 2020). The activation from the latent form in plants is unknown; nevertheless, some studies speculate that the presence of proteases, acidic environments, fatty acids, and detergents could trigger the latent state of PPO. Another possibility is the presence of endogenous substrates (Saranya et al., 2023). In this work, we have expressed the full sequence of *ppoMP* comprising the active and the C-terminal domains. To the best of our knowledge, this is the first instance of a PPO being expressed *in vivo* in *E. coli* in its active form as previous studies focused on the expression of PPOs for purification and subsequent activation *in vitro* (Panis et al., 2020; Saranya et al., 2023). In our study, the enzyme is active under the conditions tested as indicated by the accumulation of dopamine, although the activation mechanism remains unknown. On the contrary, the other PPO tested from *A. bisporus* might be in its latent form since no dopamine was detected when co-expressing this enzyme with *tdc*. In contrast to the observations made with the top-performing dopamine producer employing the known pathway (DPM15), where the intermediate compound L-DOPA was not detected, it is noteworthy that 0.7 g/L of tyramine were detected in the case of DPA2, the most effective dopamine-producing strain expressing the novel pathway (Supplementary Figure S4). This indicates a potentially significant inefficiency in enzymatic steps and suggests room for further optimization. Considering this, alongside the fact that the maximum titers achieved with the two pathways are in the same order of magnitude, underscores the promising potential of further exploring this novel dopamine production pathway. Specifically, we believe that a better understanding of PPOs function and activation as discussed above can lead to an improvement in the catalytic activity of this step.

In our production experiments, the choice of host strain and the use of specific promoters were observed to influence the production of L-DOPA and dopamine. JM109-based strains generally exhibited higher L-DOPA titers, while the decarboxylation of L-DOPA into dopamine seemed to be favored in BL21 cells. This trend was also evident in the expression of enzymes constituting the novel dopamine pathway, particularly in the initial decarboxylation of tyrosine into tyramine. Hence, it appears that decarboxylation reactions are more active in BL21-based strains compared to K12 cells. One plausible explanation could be that these strains preferentially promote the metabolism of CO_2_, potentially creating a driving force for biosynthetic pathways requiring a decarboxylation step. However, this hypothesis necessitates further investigation.

Generally, the inactivation of the regulator involved in aromatic amino acid metabolism and enzymes involved in a tyrosine competing pathway did not lead to higher titers, except for the DPM15 strain, which achieved the maximum dopamine titer expressing the known pathway in BL21 cells having the genes *pheLA* and *tyrR* deleted. The shikimate pathway, especially the tyrosine biosynthetic pathway, is highly regulated. Thus, developing a tyrosine chassis strain is a laborious task including multiple genetic modifications such as gene knock-outs, overexpression, and replacement of feedback-resistant protein motifs (Lütke-Eversloh et al., 2007; Fordjour et al., 2019). We have selected the two-point genetic modifications (Δ*pheLA* and Δ*tyrR*) to implement in our strains considering that these two knockouts were the most common modification encountered in tyrosine over-producing strains (Averesch and Krömer, 2018). The deletion of *tyrR* aims to prevent transcriptional regulation, while the deletion of *pheLA* aims to reduce the diversion of AAA pathway intermediates towards competing reactions (Santos et al., 2012). Muñoz et. al. have observed 1.9-fold increment in specific rate of tyrosine production when inactivating TyrR (Muñoz et al., 2011), while the knockout of *pheLA* led to an increment in L-DOPA production from tyrosine in the study carried out by Wei et al. (2016). However, the impact of these mutations on tyrosine accumulation and, consequently, on the production of the target compounds in our study was difficult to foresee since we are supplementing tyrosine to our strains. Nevertheless, it seems that these changes were insufficient to accumulate a high amount of tyrosine for further conversion into the target products. Direct supplementation of tyrosine aims to simulate the behavior of a chassis strain, aiding in the identification of the most efficient producer strains. However, as part of future work, it would be crucial to evaluate the biosynthetic pathways for L-DOPA and dopamine in industrial tyrosine-chassis strains.

Our findings highlight the complexity of the pathway and the various factors influencing L-DOPA and dopamine production, including the choice of genes, host strain, promoter, and cofactor supplementation. Further optimization and detailed characterization of the enzymes involved are necessary to fully leverage these pathways for L-DOPA and dopamine production. Not all implemented pathways resulted in the accumulation of target compounds, in particular, the expression of PPO from *A. bisporus* in the novel dopamine pathway. This could be attributed to the lack of activation of the enzyme, which is pivotal for these types of enzymes due to the co-expression of a terminal blocking the active site. Unlike the PPO from *M. pruriens*, the *A. bisporus* enzyme might exhibit a low activity towards the substrate or other underlying problems that require further investigation.

One approach to address metabolic bottlenecks and improve pathway performance would be the use of protein engineering methods combining protein structure prediction and molecular docking (Trott and Olson, 2010) to introduce mutations that enhance enzyme activity or affinity to the target substrate. Specifically, the novel dopamine pathway could greatly benefit from a more active second step, as tyramine accumulated at titers of 0.7 g/L in the best dopamine producer (strain DPA2). Our GDEE tool enables us to conduct experiments within the enzyme engineering domain, and we are presently exploring this prospect. Additionally, another potential strategy involves initiating a new round of gene discovery by leveraging a predicted structure for *ppoMP*, aiming to uncover new potential gene sequences for this crucial step.

Despite the availability of enumeration tools for pathway design, their utilization in previous studies has been limited, often being employed in isolation. Although various pathway enumeration tools have emerged (Hatzimanikatis et al., 2005; Liu et al., 2015; Koppolu and Vasigala, 2016; Delépine et al., 2018; Shibukawa et al., 2020; Hafner and Hatzimanikatis, 2021; Mohammadi-Peyhani et al., 2021; Sveshnikova et al., 2022a; Sveshnikova et al., 2022b), it appears that many synthetic biologists still predominantly rely on literature and database reviews, in conjunction with accumulated knowledge, for the pathway design process, as indicated by the limited citations of experimental works (Fehér et al., 2014; Tokic et al., 2018; Ferreira et al., 2019; Liu et al., 2021).

Our work demonstrates the successful integration of computational tools for pathway, enzyme and gene candidate selection, paving the way for more advanced applications, including the integration of Artificial Intelligence (AI) tools (Jang et al., 2022). Combining AI with automation of Design-Build-Test-Learn (DBTL) principles holds great promise for the automatic and robust design of microbial cell factories (Carbonell et al., 2018; HamediRad et al., 2019; Radivojević et al., 2020; Sveshnikova et al., 2022b; Liao et al., 2022; Gurdo et al., 2023). This approach is particularly relevant for complex compounds and poorly understood or inefficient metabolic pathways, as it allows for systematic exploration and optimization.

AI-guided enzyme and pathway design have the potential to expand the portfolio of biosynthetic pathways, enabling the production of both natural and synthetic compounds. The computational workflow described in this study represents a significant step forward, although it still requires manual curation and analysis. The future integration of standardized information, robotic handling platforms and machine learning methods holds the promise of fully automated pipelines with minimal human intervention. Such advancements would facilitate the rapid and efficient design of microbial cell factories, enabling the selection of the most effective variants through iterative DBTL cycles (HamediRad et al., 2019; Liao et al., 2022; Gurdo et al., 2023).

In conclusion, our study validates the use of computational tools for the design of biosynthetic pathways, showcasing their potential in the production of high-value compounds like L-DOPA and dopamine. The integration of AI and automation, along with further advancements in protein engineering, will contribute to expanding our understanding of metabolic pathways and revolutionize the field of microbial cell factory design.

## Data Availability

The original contributions presented in the study are included in the article/Supplementary Material, further inquiries can be directed to the corresponding author.
