# Supplementary material for: Computer-aided design and implementation of efficient biosynthetic pathways to produce high added-value products derived from tyrosine in Escherichia coli
Source: Front Bioeng Biotechnol. 2024 Jun 24;12:1360740. doi: 10.3389/fbioe.2024.1360740 (PMC11228882; doi:10.3389/fbioe.2024.1360740)
Supplement: Supplementary file 1 [file DataSheet1.docx]

**Supplementary material:**

***Primers***

***Table S1:*** List of primers used in this study for the different molecular biology protocols.

| ***Name*** | **Sequence (5′ to 3′)** |
| --- | --- |
| ***Gene Knockouts (underlined sequence corresponds to the N20 region)*** | |
| *gRNA_pheLA_fw:* | ATAAGGCCTCCCAAATCGGGGTTTTAGAGCTAGAAATAGCAAGTTAAAATAAG |
| *gRNA_pheLA_rev* | CCCGATTTGGGAGGCCTTATACTAGTATTATACCTAGGACTGAG |
| *gRNA_tyrR_fw* | CTGTACTTACGCGTTATGCGGTTTTAGAGCTAGAAATAGCAAGTTAAAATAAG |
| *gRNA_tyrR_rev* | CGCATAACGCGTAAGTACAGACTAGTATTATACCTAGGACTGAG |
| ***Plasmids*** | |
| ***pETDuet_MCS1_NcoI_fwd*** | GGCAGCAGCCATCAC |
| ***pETDuet_MCS1_NcoI_rev*** | CATGGTATATCTCCTTCTTAAAGTTAAAC |
| ***pETDuet_MCS2_NdeI_fwd*** | GCAGATCTCAATTGGATATC |
| ***pETDuet_MCS2_NdeI_rev*** | CATATGTATATCTCCTTCTTATACTTAAC |
| ***pETDuet_MCS1_EcoRI_fw*** | GCGGCCGCATAATGCTTAAG |
| ***pETDuet_MCS1_NotI_rev*** | GAATTCGGATCCTGGCTG |
| ***pETDuet_MCS1_XhoI_rev*** | CATATGTATATCTCCTTCTTATACTTAAC |
| ***Gene cloning***  ***(lower case – homologous sequence to the plasmid; upper case – homologous sequence to the gene)*** | |
| ***tyr_fw*** | aagaaggagatatacatatgGTTGTTCGCCGTACAGTC |
| ***tyr_rev*** | gatatccaattgagatctgcTCAGATCACCGCGACTTC |
| ***ddc_fw*** | cacagccaggatccgaattcATGACACCCGAGCAATTTC |
| ***ddc_rev*** | cttaagcattatgcggccgcTTACGAGCCTCCCTTGATTAC |
| ***tdc_fw*** | cacagccaggatccgaattcATGGAAAAATCAAACCGC |
| ***tdc_rev*** | cttaagcattatgcggccgcTTATACATTTTCTTTCTGGTTG |
| ***ppoAB_fw*** | aagaaggagatatacatatgAGCTTGATCGCCACCGTAG |
| ***ppoAB_rev*** | gatatccaattgagatctgcTTAGTTGATTACGTGTACGGCTAC |
| ***ppoMP_fw*** | aagaaggagatatacatatgGCGTCGTTATACCCCTCTC |
| ***ppoMP_rev*** | gatatccaattgagatctgcTCATTTATGAAACTCAATTTTGATACCC |
| ***Site directed mutagenesis (uppercase bps correspond to the punctual mutations)*** | |
| ***tyr_Y119F_fw*** | tagctgcacttacagggtTTaaaacttttgcaatgcc |
| ***tyr_Y119F_rev*** | ggcattgcaaaagttttAAaccctgtaagtgcagcta |
| ***tyr_V153A_fw*** | ggaaaacaaacccactttatgCccctaatcgtaacgaacttac |
| ***tyr_V153A_rev*** | gtaagttcgttacgattagggGcataaagtgggtttgttttcc |
| ***tyr_D317Y_fw*** | gacgtaatgccgcgcgccTataataaagtggttaataa |
| ***tyr_D317Y_rev*** | ttattaaccactttattAtaggcgcgcggcattacgtc |
| ***tyr_L320_fw*** | gcgtgcagagcacctgGttgcgctttttaaaac |
| ***tyr_L320_rev*** | gttttaaaaagcgcaaCcaggtgctctgcacgc |
| ***tdc_S587A_fw*** | gtgacaattattcgcgcgGctgtgatgacaccatata |
| ***tdc_S587A_rev*** | tatatggtgtcatcacagCcgcgcgaataattgtcac |
| ***Promoter switch (T7 → trc) (uppercase bps correspond to trc promoter sequence)*** | |
| pETDuet_trc1lacO_fw | CGGCTCGTATAATGggaattgtgagcggataacaattcccctctagaaataattttgtttaactttaag |
| ***pETDuet_trc1lacO_rev*** | caattccCATTATACGAGCCGGATGATTAATTGTCAAatttcgcgggatcgagatc |
| ***pETDuet_trc2lacO_fw*** | CGGCTCGTATAATGggaattgtgagcggataacaattccccatcttagtatattagttaagtataag |
| ***pETDuet_trc2lacO_rev*** | caattccCATTATACGAGCCGGATGATTAATTGTCAAatttcgattatgcggccg |

 fw – forward; rev – reverse.


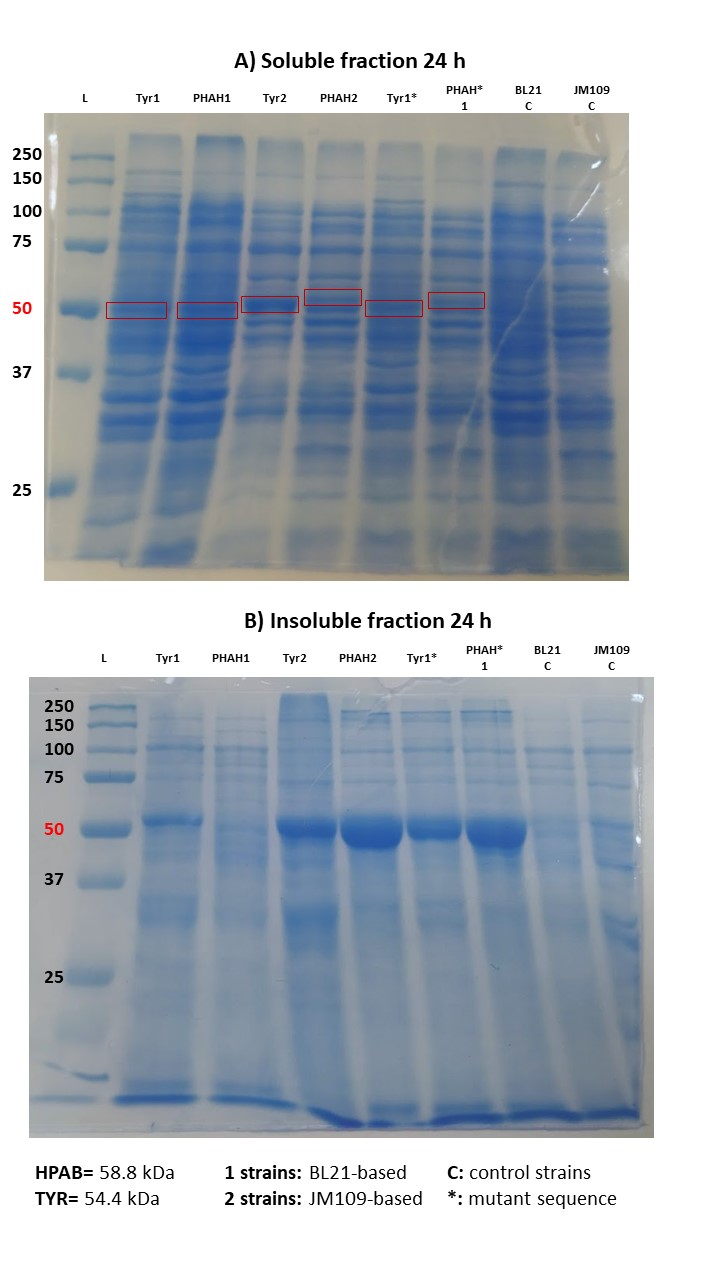
***SDS-PAGE gel***

***Figure S1:*** SDS-PAGE analysis of crude extracts of *Escherichia coli* strains to yield L-DOPA expressing either p-hydroxyphenylacetate 3-hydroxylase (PHAH), codified by the *hpaBC* gene from *E. coli*, or Tyrosinase (*tyr* from *Ralstonia solanacearum*) in BL21 cells (1) or JM109 cells (2). Control lanes correspond to cells without the plasmids. A) Soluble fraction 24 hours after IPTG induction; B) insoluble fraction after 24 hours of induction. The expected size for *hpaB* is 58.8 kDa and for *tyr* is 54.5 kDa. *: mutant sequences (*tyr** Y119F | V153A | D317Y | L330V; *hpaBC**= G295R).

***NMR spectra
-LDOPA (LD) and Dopamine known pathway (DPM) strains***

***
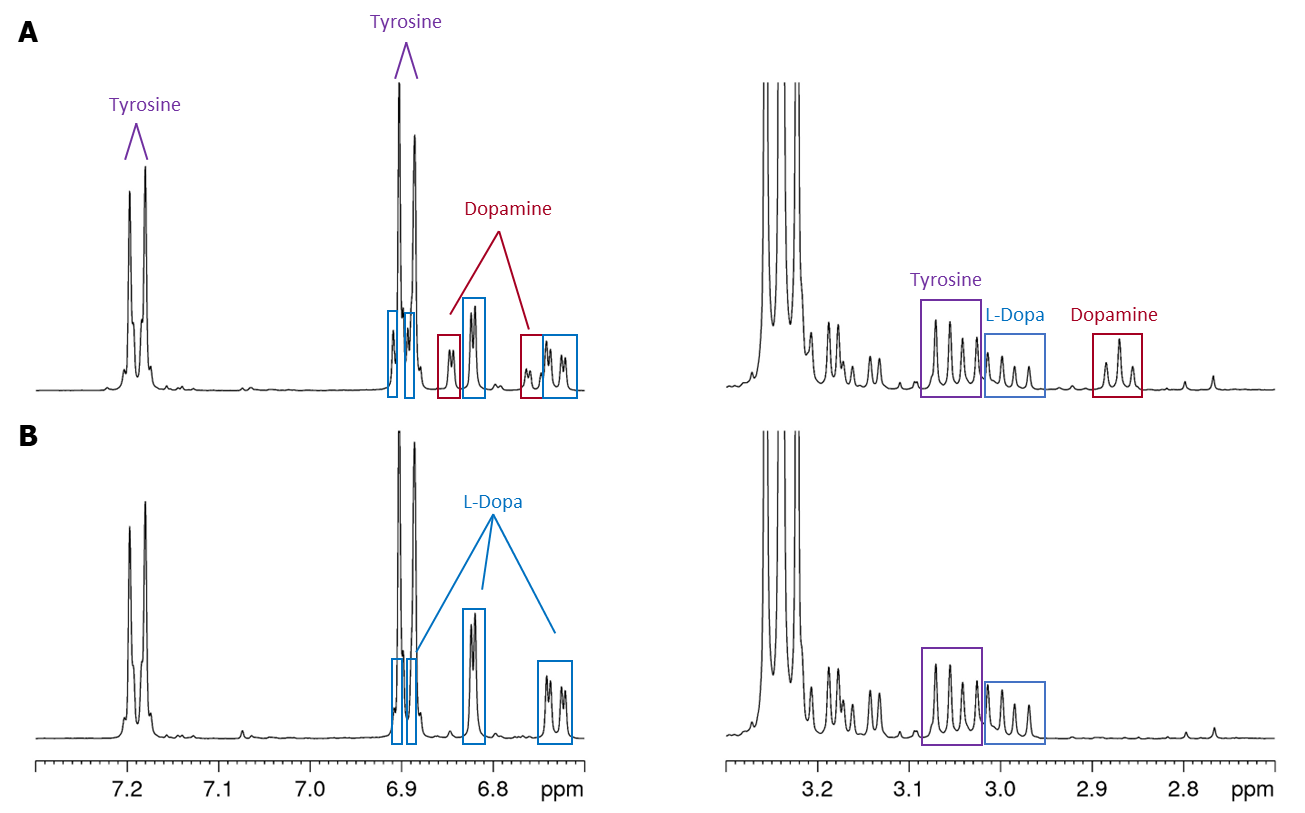
***

***Figure S2:*** ^1^H-NMR spectra highlighting A) samples for DPM strains and B) samples for LD strains, with the resonances corresponding to L-DOPA, dopamine, and tyrosine indicated. The production of L-DOPA is achieved by expressing a mutant sequence of tyrosinase (*tyr*) from *Ralstonia solanacearum*, which converts tyrosine into L-DOPA, and extending the pathway by expressing *ddc* from *Pseudomonas putida* allows for dopamine accumulation.

***
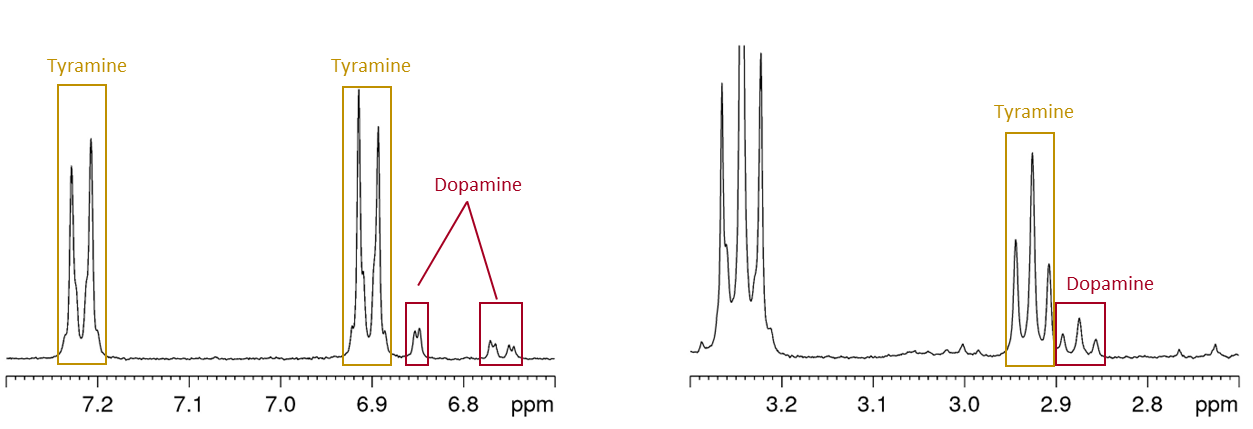
-Dopamine novel pathway (DPA) strains
Figure S3:*** ^1^H-NMR spectra highlight showing the production of dopamine in DPA strains. The resonances due to dopamine and tyramine are indicated. This pathway offers an alternative method to produce dopamine from tyrosine. First, tyrosine decarboxylase (*tdc*) from *Levilactobacillus brevis* converts tyrosine to tyramine, which is then transformed into dopamine by expressing polyphenol oxidase (*ppo*) from *Mucuna pruriens*.

***Tyramine Titers***

***
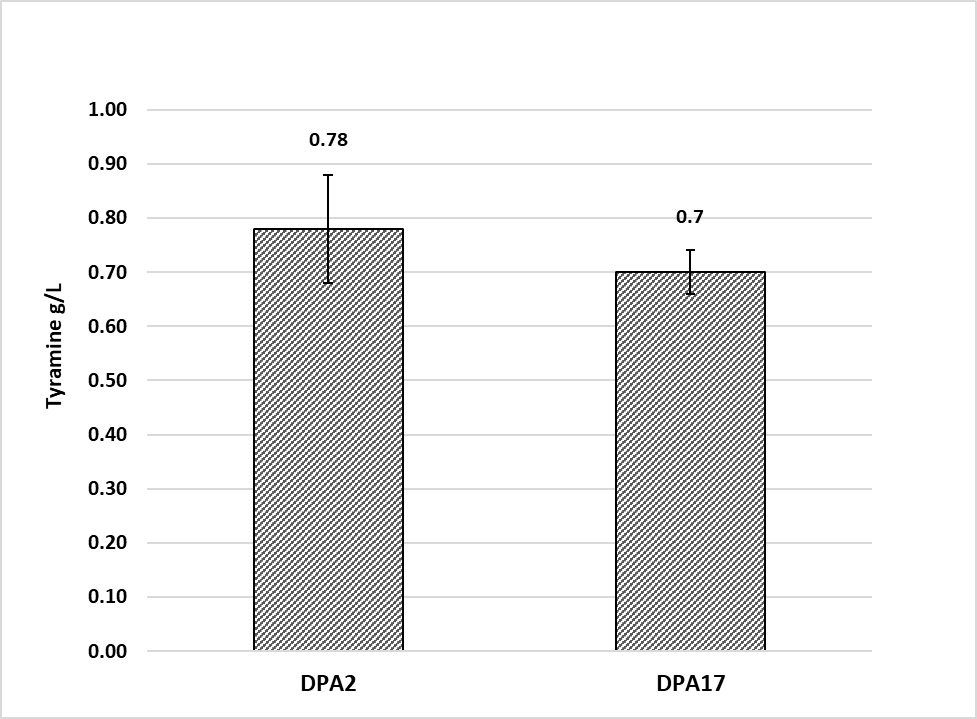
***

**Figure S4:** Tyramine production for DPA2 and DPA17 strains. This pathway offers an alternative method to produce dopamine from tyrosine. First, tyrosine decarboxylase (tdc) from Levilactobacillus brevis converts tyrosine to tyramine, which is then transformed into dopamine by expressing polyphenol oxidase (ppo) from Mucuna pruriens.

**Table Enzyme Selection**

**Table S2**: List of the Enzyme Commission (EC) numbers attributed by Selenzyme and BridgIT to the different reactions constituting the pathways described in this work. For each reaction, the different EC numbers attributed, and respective Reaction Similarity Score (from Selenzyme) are listed. The Reaction Similarity Score represent the probability of an enzyme belonging to a specific EC number catalyze the input reaction. Only EC numbers with Reaction Similarity score equal to one were considered for implementing the Gene Discovery step in the Gene Discovery and Enzyme Engineering pipeline.

| Reaction | EC number | Description | Reaction Similarity Score |
| --- | --- | --- | --- |
| *Tyrosine → L-DOPA* | 1.14.14 | 4-hydroxyphenylacetate 3-monooxygenase | **1** |
|  | 1.14.16.2 | Tyrosine 3-monooxygenase | **1** |
|  | 1.10.3.1; 1.14.18.1; | Tyrosinase | **1** |
| *L-DOPA → Dopamine* | 4.1.1.25; 4.1.1.28 | Aromatic-L-amino-acid/ DOPA decarboxylase | **1** |
|  | 2.1.1.6 | Catechol O-methyltransferase | 0.80477 |
|  | 1.14.17.1 | Dopamine beta-hydroxylase | 0.79828 |
| *Tyrosine → Tyramine* | 4.1.1.25;  4.1.1.28 | L-tyrosine decarboxylase | **1** |
|  | 1.14.13.41 | Tyrosine N-monooxygenase | 0.83897 |
|  | 4.1.99.19 | 2-iminoacetate synthase | 0.81955 |
| *Tyramine → Dopamine* | 1.14.17 | L-tyrosine decarboxylase | 0.79569 |
|  | 2.1.1.6 | Catechol O-methyltransferase | 0.79440 |
|  | 1.4.3.21;  1.4.3.4;  1.4.9.2 | Amine oxidase [flavin-containing] A | 0.79435 |
|  | 1.14.17.1 | Dopamine beta-hydroxylase | 0.78149 |
|  | 4.1.1.25;  4.1.1.28 | L-tyrosine decarboxylase | 0.77691 |

**Table S3**: Top-ranked list of various enzyme sequences and their respective Enzyme Commission (EC) numbers attributed by Selenzyme to the diverse reactions constituting the pathways described in this work. For each reaction constituting the different biosynthetic pathways, the top selected sequences are listed based on the predefined combined score. The score considers reaction similarity, phylogenetic distance, UniProt protein evidence, and sequence conservation. The respective UniProt ID, source organism, and EC numbers are listed.

| Reaction | Name | SEQ ID | Organism | EC number | Score |
| --- | --- | --- | --- | --- | --- |
| Tyrosine →  L-DOPA | 4-hydroxyphenylacetate 3-monooxygenase oxygenase | Q57160 | *Escherichia coli* | 1.14.14 | **97.900** |
|  | Tyrosinase | P33180 | *Rhizobium meliloti* | 1.10.3.1;1.14.18.1 | 86.700 |
|  | Tyrosinase | P06845 | *Streptomyces glaucescens* | 1.10.3.1;1.14.18.1 | 84.900 |
|  | Tyrosinase | P55022 | *Streptomyces galbus* | 1.10.3.1;1.14.18.1 | 84.700 |
|  | Tyrosinase | P55023 | *Streptomyces lincolnensis* | 1.10.3.1;1.14.18.1 | 84.700 |
|  | Tyrosinase | P07524 | *Streptomyces antibioticus* | 1.10.3.1;1.14.18.1 | 84.700 |
| L-DOPA → Dopamine | Probable aromatic-L-amino-acid decarboxylase | P34751 | *Caenorhabditis elegans* | 4.1.1.25;4.1.1.28 | **75.800** |
|  | Tyrosine/DOPA decarboxylase 2 | P54769 | *Papaver somniferum* | 4.1.1.25;4.1.1.28 | 73.800 |
|  | Tyrosine/DOPA decarboxylase 3 | P54770 | *Papaver somniferum* | 4.1.1.25;4.1.1.28 | 73.800 |
|  | Aromatic-L-amino-acid decarboxylase | P17770 | *Catharanthus roseus* | 4.1.1.25;4.1.1.28 | 68.800 |
|  | TynB | D7F5L7 | *Pseudomonas putida* | 4.1.1.25;4.1.1.28 | 68.251 |
|  | TynA | D7F5L6 | *Pseudomonas putida* | 4.1.1.25;4.1.1.28 | 68.251 |
| Tyrosine  → Tyramine | L-tyrosine decarboxylase | Q60358 | *Methanocaldococcus jannaschii* | 4.1.1.25;4.1.1.28 | **82.900** |
|  | L-tyrosine decarboxylase | A7IAB9 | *Methanoregula boonei* | 4.1.1.25;4.1.1.28 | 82.700 |
|  | L-tyrosine decarboxylase | B0R349 | *Halobacterium salinarum* | 4.1.1.25;4.1.1.28 | 82.700 |
|  | L-tyrosine decarboxylase | Q3IT46 | *Natronomonas pharaonis* | 4.1.1.25;4.1.1.28 | 82.700 |
|  | L-tyrosine decarboxylase | Q2FSD2 | *Methanospirillum hungatei JF-1* | 4.1.1.25;4.1.1.28 | 82.700 |
|  | L-tyrosine decarboxylase | Q0W498 | *Methanocella arvoryzae* | 4.1.1.25;4.1.1.28 | 82.700 |
| Tyramine  →  Dopamine | TynA | D7F5L6 | *Pseudomonas putida* | 1.4.3.21;1.4.3.22;1.4.3.4;  1.4.9.2 | **67.883** |
|  | TynB | D7F5L7 | *Pseudomonas putida* | 1.4.3.21;1.4.3.22;1.4.3.4;  1.4.9.2 | 67.883 |
|  | L-tyrosine decarboxylase | Q60358 | *Methanocaldococcus jannaschii* | 4.1.1.25;4.1.1.28 | 60.591 |
|  | L-tyrosine decarboxylase | Q0W498 | *Methanocella arvoryzae* | 4.1.1.25;4.1.1.28 | 60.391 |
|  | L-tyrosine decarboxylase | C5A2X8 | *Thermococcus gammatolerans* | 4.1.1.25;4.1.1.28 | 60.391 |
|  | L-tyrosine decarboxylase | A2STQ3 | *Methanocorpusculum labreanum* | 4.1.1.25;4.1.1.28 | 60.391 |

**Tables GDEE

Table S4:** All 24 candidate enzymes selected for the production of L-DOPA from Tyrosine after filtering the BLAST results, organized by their binding affinity score (obtained by Autodock Vina). The Uniprot ID of the enzyme used as a template is marked with an asterisk.

| **Rank** | **Uniprot ID** | **ΔG (kcal/mol)** | **Organism** |
| --- | --- | --- | --- |
| 1 | Q9MB14 | -6.7 | *Ipomoea batatas* |
| 2 | Q08296 | -6.6 | *Solanum lycopersicum* |
| 3 | Q08305 | -6.4 | *Solanum lycopersicum* |
| 4 | Q08303 | -6.4 | *Solanum lycopersicum* |
| 5 | Q06355 | -6.4 | *Solanum tuberosum* |
| 6 | Q8Y2J8* | -6.1 | *Ralstonia solanacearum* |
| 7 | O81103 | -6.1 | *Prunus armeniaca* |
| 8 | Q06215 | -6.0 | *Vicia faba* |
| 9 | P43311 | -6.0 | *Vitis vinifera* |
| 10 | H2A0L1 | -5.9 | *Margaritifera margaritifera* |
| 11 | C7FF05 | -5.9 | *Agaricus bisporus* |
| 12 | Q08304 | -5.9 | *Solanum lycopersicum* |
| 13 | Q08306 | -5.9 | *Solanum lycopersicum* |
| 14 | Q00024 | -5.8 | *Agaricus bisporus* |
| 15 | Q6UIL3 | -5.8 | *Larrea tridentata* |
| 16 | O42713 | -5.7 | *Agaricus bisporus* |
| 17 | Q10583 | -5.7 | *Megathura crenulata* |
| 18 | Q92396 | -5.7 | *Podospora anserina* |
| 19 | Q9FRX6 | -5.7 | *Antirrhinum majus* |
| 20 | P43310 | -5.7 | *Spinacia oleracea* |
| 21 | O61363 | -5.6 | *Enteroctopus dofleini* |
| 22 | C7FF04 | -5.6 | *Agaricus bisporus* |
| 23 | P81732 | -5.5 | *Megathura crenulata* |
| 24 | P00440 | -5.5 | *Neurospora crassa* |

**Table S5**: All 64 candidate enzymes selected for the transformation of L-DOPA into Dopamine after filtering the BLAST results, organized by their binding affinity score (obtained by Autodock Vina). The Uniprot ID of the enzyme used as a template is marked with an asterisk.

| **Rank** | **Uniprot ID** | **ΔG (kcal/mol)** | **Organism** |
| --- | --- | --- | --- |
| 1 | P54769 | -7.2 | *Papaver somniferum* |
| 2 | Q9M0G4 | -7 | *Arabidopsis thaliana* |
| 3 | Q05733 | -6.7 | *Drosophila melanogaster* |
| 4 | Q0ZS27 | -6.7 | *Rosa hybrid cultivar* |
| 5 | Q6ZJK7 | -6.6 | *Oryza sativa subsp. japonica* |
| 6 | Q8RY79 | -6.6 | *Arabidopsis thaliana* |
| 7 | P17770 | -6.6 | *Catharanthus roseus* |
| 8 | Q06087 | -6.5 | *Petroselinum crispum* |
| 9 | A0A2H5AIY0 | -6.5 | *Narcissus pseudonarcissus* |
| 10 | Q94EE9 | -6.4 | *Oryza sativa subsp. japonica* |
| 11 | P54771 | -6.4 | *Papaver somniferum* |
| 12 | P93083 | -6.4 | *Camptotheca acuminata* |
| 13 | Q06085 | -6.3 | *Petroselinum crispum* |
| 14 | O96569 | -6.3 | *Drosophila lebanonensis* |
| 15 | P54768 | -6.3 | *Papaver somniferum* |
| 16 | Q06088 | -6.2 | *Petroselinum crispum* |
| 17 | Q0ZQX0 | -6.2 | *Petunia hybrida* |
| 18 | P93082 | -6.2 | *Camptotheca acuminata* |
| 19 | P34751 | -6.1 | *Caenorhabditis elegans* |
| 20 | Q64611 | -6.1 | *Rattus norvegicus* |
| 21 | Q06086 | -6.1 | *Petroselinum crispum* |
| 22 | Q99K01 | -6 | *Mus musculus* |
| 23 | Q9Y600 | -6 | *Homo sapiens* |
| 24 | Q95ZS2 | -6 | *Caenorhabditis elegans* |
| 25 | P48319 | -6 | *Sus scrofa* |
| 26 | O82415 | -6 | *Papaver somniferum* |
| 27 | P16453 | -5.9 | *Rattus norvegicus* |
| 28 | Q16S21 | -5.8 | *Aedes aegypti* |
| 29 | A0A2I6B3P0 | -5.8 | *Rhodiola rosea* |
| 30 | Q9DBE0 | -5.8 | *Mus musculus* |
| 31 | P23738 | -5.8 | *Mus musculus* |
| 32 | Q9MA74 | -5.7 | *Arabidopsis thaliana* |
| 33 | Q7XHL3 | -5.7 | *Oryza sativa subsp. japonica* |
| 34 | P54770 | -5.7 | *Papaver somniferum* |
| 35 | P05031 | -5.6 | *Drosophila melanogaster* |
| 36 | P14748 | -5.6 | *Felis catus* |
| 37 | A0PA85 | -5.6 | *Canis lupus familiaris* |
| 38 | Q88JU5* | -5.4 | *Pseudomonas putida* |
| 39 | Q5E6F9 | -5.4 | *Aliivibrio fischeri* |
| 40 | O88533 | -5.3 | *Mus musculus* |
| 41 | P48318 | -5 | *Mus musculus* |
| 42 | A7B1V0 | -4.8 | *Ruminococcus gnavus* |
| 43 | Q5R7S7 | -4.8 | *Pongo abelii* |
| 44 | P20228 | -4.8 | *Drosophila melanogaster* |
| 45 | P20711 | -4.6 | *Homo sapiens* |
| 46 | P18088 | -4.6 | *Rattus norvegicus* |
| 47 | Q0VCA1 | -4.6 | *Bos taurus* |
| 48 | Q5EA83 | -4.5 | *Bos taurus* |
| 49 | Q99259 | -4.5 | *Homo sapiens* |
| 50 | A0A2H5AIY2 | -4.5 | *Narcissus pseudonarcissus* |
| 51 | P48861 | -4.4 | *Manduca sexta* |
| 52 | P18486 | -4.2 | *Drosophila melanogaster* |
| 53 | Q43908 | -4.2 | *Acinetobacter baumannii* |
| 54 | P14173 | -4.1 | *Rattus norvegicus* |
| 55 | Q5IS68 | -4 | *Pan troglodytes* |
| 56 | Q28D99 | -3.9 | *Xenopus tropicalis* |
| 57 | P22781 | -3.8 | *Cavia porcellus* |
| 58 | Q9Z3R1 | -3.4 | *Rhizobium meliloti* |
| 59 | P19113 | -3.3 | *Homo sapiens* |
| 60 | A0A0A2IDH4 | -3.3 | *Penicillium expansum* |
| 61 | P80041 | -3.1 | *Sus scrofa* |
| 62 | O96571 | -2.7 | *Drosophila lebanonensis* |
| 63 | P27718 | -2.6 | *Bos taurus* |
| 64 | O96567 | -0.6 | *Drosophila simulans* |

- **Effect of acid ascorbic supplementation**

**
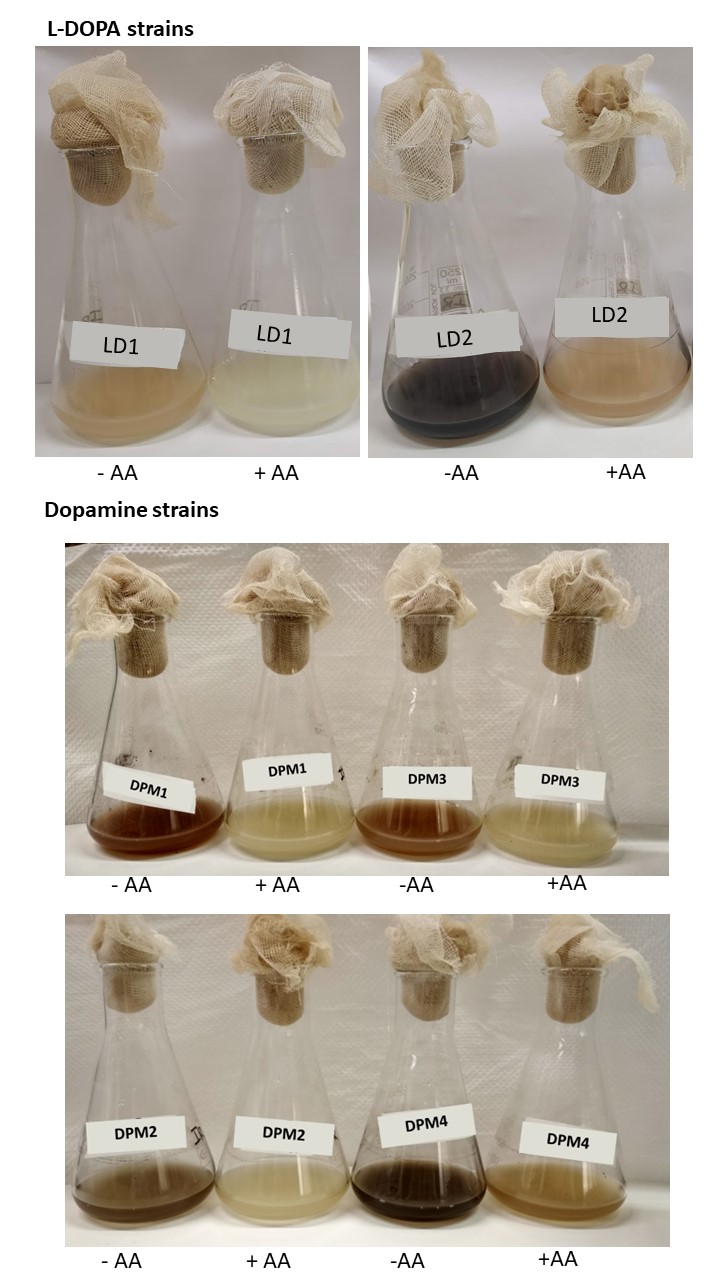
**

***Figure S5:*** Effect of ascorbic acid (AA) supplementation on the media color for LD (L-DOPA) and DPM (Dopamine) known pathway-producing strains. The production of L-DOPA is achieved by expressing a mutant sequence of tyrosinase (*tyr*) from *Ralstonia solanacearum*, which converts tyrosine into L-DOPA, and extending the pathway by expressing *ddc* from *Pseudomonas putida* allows for dopamine accumulation.
